# Gut Microbiota: Influence on Carcinogenesis and Modulation Strategies by Drug Delivery Systems to Improve Cancer Therapy

**DOI:** 10.1002/advs.202003542

**Published:** 2021-03-09

**Authors:** Runqi Zhu, Tianqun Lang, Wenlu Yan, Xiao Zhu, Xin Huang, Qi Yin, Yaping Li

**Affiliations:** ^1^ State Key Laboratory of Drug Research and Center of Pharmaceutics Shanghai Institute of Materia Medica Chinese Academy of Sciences 501 Haike Road Shanghai 201203 China; ^2^ School of Pharmacy University of Chinese Academy of Sciences Beijing 100049 China; ^3^ Yantai Key Laboratory of Nanomedicine and Advanced Preparations Yantai Institute of Materia Medica Yantai 264000 China; ^4^ School of Pharmacy Yantai University Yantai 264005 China

**Keywords:** cancer therapy, carcinogenesis, drug delivery system, gut microbiota, modulation

## Abstract

Gut microbiota have close interactions with the host. It can affect cancer progression and the outcomes of cancer therapy, including chemotherapy, immunotherapy, and radiotherapy. Therefore, approaches toward the modulation of gut microbiota will enhance cancer prevention and treatment. Modern drug delivery systems (DDS) are emerging as rational and promising tools for microbiota intervention. These delivery systems have compensated for the obstacles associated with traditional treatments. In this review, the essential roles of gut microbiota in carcinogenesis, cancer progression, and various cancer therapies are first introduced. Next, advances in DDS that are aimed at enhancing the efficacy of cancer therapy by modulating or engineering gut microbiota are highlighted. Finally, the challenges and opportunities associated with the application of DDS targeting gut microbiota for cancer prevention and treatment are briefly discussed.

## Graphical abstract

Gut microbiota play essential roles in carcinogenesis, cancer progression, and cancer therapy. Modern drug delivery systems (DDS) have been exploited as rational and promising tools for microbiota modulation. DDS are able to overcome obstacles associated with traditional microbiota‐modulating treatments and efficiently improve therapeutic effects on cancer by delivering prebiotics and probiotics, selectively eliminating pro‐tumoral bacteria, and capturing bacteria products.

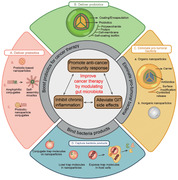

## 1 Introduction

The human body ecosystem contains a huge reservoir of microbes that perform important functions. Microbiota refer to the collection of commensal, symbiotic, and pathogenic microorganisms including bacteria, archaea, protists, fungi, and viruses that form an ecological community in and on multicellular organisms. The totality of these microbial communities, including their genetic information and the milieu they inhabit, is termed as microbiome. Microbiome and the host are studied as synergistic units at the epigenetic and genomic level. In the human body, the clustering patterns of microbiota are remarkably different across body locations and among individuals. This clustering has not been fully elucidated.^[^ ^1^ ^]^ In particular, the human gut, as a system with large surface area and nutrient‐rich environment, is colonized by about 100 trillion microbes that outnumber the body cells by up to tenfold and can be categorized into an estimated more than 1000 species.^[^ ^2^ ^]^ The microbiota inhabit the gastrointestinal tract (GIT), as the most studied human microbiota, influences the normal physiology and the development of diseases through its collective host interactions in digestion, nutrition absorption, metabolic activities, and immune responses.^[^ ^3^ ^]^ In the human gene landscape, gut microorganisms provide more gene material than human body cells by a couple of magnitudes.^[^ ^4^ ^]^ In view of evolution, the human body and microbiome are regarded as a superorganism due to their close relationships.^[^ ^5^ ^]^ Gut microbiota can be shaped by a series of concurrent factors and respond to environmental changes inside and outside the body.^[^ ^6^ ^]^ It has been reported that dietary habits,^[^ ^7^ ^]^ lifestyles,^[^ ^8^ ^]^ physiological states,^[^ ^9^ ^]^ genetics,^[^ ^10^ ^]^ and even age^[^ ^11^ ^]^ are all responsible for the composition of gut microbiota. Furthermore, pathogenesis, carcinogenesis, and administration of therapeutic agents affect the characteristics of the microbiome community.^[^ ^12^ ^]^

Recent advancements in microbiota analysis have elucidated on the human‐microbiota interplay.^[^ ^13^ ^]^ High‐throughput genome sequencing, also known as next‐generation sequencing of Sanger sequencing, has improved the speed of taxonomic identification.^[^ ^14^ ^]^ The 16S rRNA sequencing is utilized for bacterial species identification, while shotgun sequencing enables metagenomic analysis to decipher the genetic code behind the distinctive functions of human gut microbiota.^[^ ^15^ ^]^ However, one of the limitations of genomic technologies is the failure to easily detect minority populations. This failure can be addressed by culture‐dependent approaches.^[^ ^16^ ^]^ Culturomics combines multiple culture conditions and rapid identification of hundreds of new species, providing exciting perspectives on “unknown” commensal species and discovering new therapeutics.^[^ ^17^ ^]^ Due to the challenges associated with culturing and analyzing human‐microbiota complexity, “meta‐omics” approaches including gene sequencing, metagenomics, metatranscriptomics, metaproteomics, and metabolomics have been developed to study host–microbiome interactions based on genes, transcripts, proteins, and metabolites.^[^ ^4^ ^]^ Meta‐omics approaches are able to collect information for strain‐level identification, transcription, and expression activity as well as specific metabolite production. They are efficient methods for promoting the systematic understanding of microbiome classification and function.^[^ ^18^ ^]^ Microbiome‐wide association studies mentioned above have revealed the comprehensive connection between microbiome and specific diseases. In addition, to move from correlation to causal identification, an elaborate and experimentally efficient system should be established.^[^ ^19^ ^]^ The application of gnotobiotic animals can be a choice. Gnotobiotic animals are born and reared in aseptic conditions. Therefore, these animals are germ free or only contain certain known bacterial species. Due to the well‐known status of their microbial communities, gnotobiotic animals provide suitable platforms for studying symbiotic interactions related to specific diseases or physiological conditions of the human host. Human‐microbiota‐associated gnotobiotic animals are widely used to study the causal relationships between the human microbiota and diseases.^[^ ^20^ ^]^ Nevertheless, their capacity for representing the complex situations in human GIT has been questioned.^[^ ^21^ ^]^

Studies have shown that microbiota play a crucial role in human physiological functions including nutrient absorption,^[^ ^22^ ^]^ intestinal intrinsic neural networks regulation,^[^ ^23^ ^]^ intestinal barrier maintenance,^[^ ^24^ ^]^ metabolism,^[^ ^25^ ^]^ immunity, and inflammation.^[^ ^26^ ^]^ Dysbiosis and eubiosis have been used to describe the imbalance or balance of normal microbial communities, respectively. Dysbiosis refers to the abnormal changes in the number of normally dominating species and normally outcompeted species. Therefore, dysbiosis leads to the destruction of homeostasis in host–microbiota interactions, which acts as a trigger as well as a biomarker for pathogenesis.^[^ ^27^ ^]^ Studies have established that microbiota can promote the development of various diseases in the human body. Since microbial diversity is vast in the GIT, microbiota have been associated with a series of digestive system diseases, including inflammatory bowel disease (IBD),^[^ ^28^ ^]^ irritable bowel syndrome,^[^ ^29^ ^]^ chronic diarrhea,^[^ ^30^ ^]^ pancreatitis,^[^ ^31^ ^]^ and digestive system cancer.^[^ ^32^ ^]^ In addition, neuroinflammation^[^ ^33^ ^]^ and metabolic diseases^[^ ^34^ ^]^ have also been associated with microbiota abnormality. Therefore, microbiome might be a promising therapeutic target for a variety of diseases, including cancer.^[^ ^35^ ^]^ In addition, gut microbiota attract more attention compared to other human microbiota. *Helicobacter pylori* (*H. pylori*) and *Fusobacterium nucleatum* (*F. nucleatum*) are two well‐known pro‐tumoral bacteria involved in the progression of gastric cancer and colorectal cancer (CRC), respectively.^[^ ^32^, ^36^ ^]^

The fine line between health and carcinogenesis can be towed by commensal bacteria in the GIT through a variety of mechanisms, including the regulation of immunity and inflammation, interfering with metabolism, as well as mediating cell proliferation and death.^[^ ^37^ ^]^ Recently, Song et al. revealed that tumor‐associated bacteria (TAB) mediates carcinogenesis at both local and distal sites.^[^ ^38^ ^]^ However, it has not been established how gastrointestinal bacteria or their metabolites affect the non‐gut tumor microenvironment.^[^ ^39^ ^]^ Studies have documented that gut microbiota can affect the outcome of cancer therapy, especially immunotherapy^[^ ^40^ ^]^ and chemotherapy.^[^ ^41^ ^]^ Therefore, approaches aimed at targeting gut microbiota will enhance cancer therapy. Traditional modalities for microbiota modulation include fecal microbiota transplants (FMT),^[^ ^42^ ^]^ antibiotics, diet,^[^ ^43^ ^]^ pre‐, pro‐, and synbiotics.^[^ ^44^ ^]^ However, these modalities are limited by some drawbacks (e.g., lack of selectivity for specific sites or bacterial species and clinical safety concerns).^[^ ^44^ ^]^ Modern drug delivery systems (DDS) are emerging as rational and promising tools for microbiota intervention. These DDS have compensated for the limitations associated with traditional modalities. Advances in material science and engineering have facilitated the efficient delivery of pre‐, pro‐, and synbiotics.^[^ ^45^ ^]^ Nanotechnologies enable therapeutic agents aimed at microbiota modulation to reach local and distal tumor sites by virtue of reasonably selected materials, surface modification, stimuli‐responsive drug release, etc.^[^ ^38^ ^]^ Various bioinspired materials (e.g., phages,^[^ ^46^ ^]^ bacteria extracellular vesicles (EVs),^[^ ^47^ ^]^ cell membranes^[^ ^48^ ^]^) are also promising in enhancing the performance of therapeutics targeting microbiota for cancer therapy.

In this review, we first summarize the mechanisms by which commensal bacteria or microbiota dysbiosis promote cancer development and how microbiota modulate host responses to cancer therapy. Then, we elucidate on the traditional approaches and modern DDS aimed at enhancing the efficacy of cancer therapy by introducing vital works. Finally, the challenges and opportunities for the application of modern DDS targeting gut microbiota for cancer prevention and treatment are briefly discussed.

## 2 The Role of Gut Microbiota in Cancer Development

**Figure 1:**
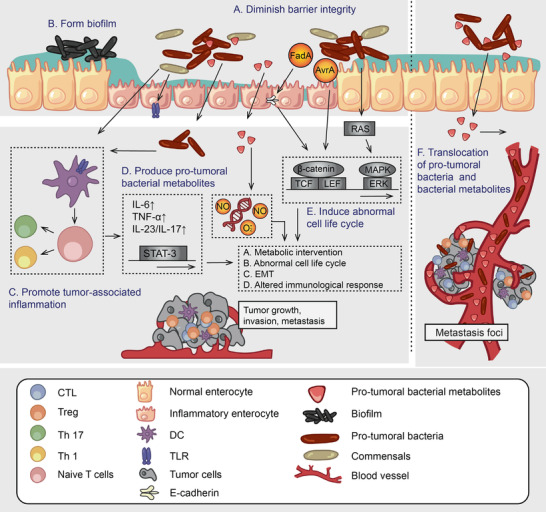
The role played by pro‐tumoral bacteria in cancer development in both local and distal sites. A) Diminish the intestinal barrier integrity. Barrier breach facilitates bacterial colonization and invasion, biofilm formation, as well as persistent inflammation. B) Form biofilms which enhances the persistent and refractory bacterial infection as well as inflammation. C) Promote tumor‐associated inflammation. Chronic inflammation caused by persistent infection mediated by NF‐kB and STAT3 signaling favor carcinogenesis. D) Produce pro‐tumoral bacterial metabolites, for example, colibactin, H_2_S, and polyamines. E) Induce abnormal cell life cycle. Bacterial products mediate cell life cycle‐associated signaling pathways including NF‐*κ*B, *β*‐catein/Wnt, RAS/MAPK. F) Tumor‐associated bacteria (TAB) mediate carcinogenesis at both local and distal sites due to the translocation of bacteria or bacterial metabolites. Based on the role played by pro‐tumoral bacteria in cancer development, eliminating pro‐tumoral bacteria and bacterial products can be rational strategies for microbiota modulation.

Cancer has become a primary threat to people's health in the modern society. It is estimated that there were 18.1 million new cancer cases and 9.6 million cancer‐associated mortalities in 2018.^[^ ^49^ ^]^ Although the causes of malignancies and metastasis have not been completely elucidated, it is widely accepted that they are as a result of anomalous interactions between the human body and genetic as well as environmental factors. The microbiome, as a special “organ’’ that is shaped by those factors, has gained much attention due to its double‐sided role as health promoter or troublemaker. The normal flora and opportunistic pathogens in GIT not only influence gut‐associated lymphoid tissue (GALT) and local metabolic functions, but also act as modulators of systemic immunity, inflammation, and metabolism. Therefore, it is not surprising that microbial communities are involved in cancer development, especially of adenocarcinomas in the GIT, through various mechanisms. In this chapter, three ways by which gut microbiota contribute to cancer development including i) influencing immunity and inflammation, ii) changing cell proliferation and death, and iii) modulating metabolism are elucidated (**Figure** **1**).

### 2.1 Microbial Immunomodulation: Proinflammatory and Immunosuppressive Responses

Immune system disorders can trigger cancer development. Immunomodulatory bacteria have been implicated in the maintenance or destruction of immune homeostasis, thereby, mediating tumorigenesis and cancer prognosis.

Since GIT is exposed to enormous dietary and other commensal antigens, gut homeostasis must be maintained by immune tolerance to these antigens along with proper immune responses to detrimental pathogens. Unregulated responses cause chronic or acute inflammation, which can cause malignant lesions. Several studies have shown a strong association between intestinal infections (e.g., IBD, Crohn's disease, and colitis) and CRC.^[^ ^27^, ^50^ ^]^ *F*. *nucleatum*, which is a carcinogenesis‐associated pathogen due to its role in promoting inflammation and suppressing adaptive immunity,^[^ ^36^ ^]^ has been studied as a target for CRC therapy. On the other hand, the immunosuppressive cancer microenvironment also enhances its progression, which means that local immune responses restricted to tumor sites are necessary. Therefore, specific immune responses against the pathogen and carcinogen are needed.

Biofilms, consisting of extracellular polymeric substance matrix enclosing multi‐microorganism aggregation,^[^ ^51^ ^]^ have been proved to favor the long‐term colonization, resistance to chemical disinfectants, and elevated virulence of bacteria adhering to biotic surfaces (e.g., GIT, lung and oral cavity mucosa).^[^ ^52^ ^]^ The formation of biofilms benefits the persistent and refractory bacterial infection as well as inflammation.^[^ ^53^ ^]^ Although chronic infection is not a typical carcinogenic factor, it is associated with up to 20% of cancers.^[^ ^54^ ^]^ Therefore, it is rational to link bacterial biofilm formation and cancer development, especially malignancies in the digestive system. Research on morphological and functional characteristics of in vitro and in vivo biofilm models by Rizzato et al. provided evidence for the hypothesis that biofilms are implicated in cancer development.^[^ ^53b^ ^]^ Colonization‐supportive traits such as low growth rates and protective metabolism shifts of *H. pylori* in biofilms have been reported.^[^ ^52^, ^53^ ^]^ Horizontal gene transfers in biofilms have also enhanced bacterial virulence and promoted inflammation.^[^ ^53^ ^]^ Dejea et al. found that bacterial biofilms could be a risk for CRC development due to their roles in upregulating epithelial cell IL‐6 and STAT3 activation and impairing epithelial barriers.^[^ ^55^ ^]^

Excessive innate immune responses can induce the production of elevated levels of chemokines and cytokines, thereby enhancing tumor‐associated inflammation. Toll‐like receptors (TLRs) and NOD‐like receptors (NLRs), as two major categories of pattern recognition receptors (PRRs), are able to detect and mediate responses to bacterial antigens (e.g., lipopolysaccharide [LPS], CpG DNA, and flagellin). TLRs signals activate transcription factors, including nuclear factor‐*κ*B (NF‐*κ*B) and activator protein 1, as a major trigger of proinflammatory responses to bacterial pathogens.^[^ ^32^, ^56^ ^]^ The innate immune system produces various proinflammatory chemokines and cytokines, including interleukin‐6 (IL‐6), tumor necrosis factor (TNF), interferon (IFN ), chemokine (C‐C motif) ligand 2, and chemokine (C‐X‐C motif) 8 (CXCL8). IL‐6/JAK/STAT3 is a primary pro‐oncogenic inflammatory pathway.^[^ ^57^ ^]^ However, signal pathways mediated by specific TLRs subsets also protect epithelium barriers in response to bacterial pathogens in order to balance mucosal homeostasis.^[^ ^58^ ^]^ Moreover, lactic acid bacteria species can induce IFN‐I release independently on PRR.^[^ ^59^ ^]^ An et al. demonstrated that sphingolipids from *Bacteroides fragilis (B. fragilis)* participated in maintaining host invariant natural killer T (iNKT) cell homeostasis in lamina propria and resulted in decreased susceptibility of specific pathogen free (SPF) mice to iNKT associated, oxazolone‐induced colitis.^[^ ^60^ ^]^

Microbe‐induced immune disorders in the GIT can also have a deleterious effect on non‐GIT organs and tissues, thereby enhancing carcinogenesis in those sites.^[^ ^37b^ ^]^ Inflammation enhances permeability of GIT epithelial barriers and promotes the access of microbes and their products to the bloodstream, which causes systematic inflammation.^[^ ^37^ ^]^ Since the liver receives most of the blood from the portal vein, the translocation of bacteria, their active metabolites, and antigens induce significant hepatic injuries and mediate hepatocarcinogenesis.^[^ ^32c^ ^]^ Gut microbiota also have been proven to influence the tumor microenvironment of pancreatic adenocarcinoma and patient survivorship.^[^ ^31^ ^]^ In addition, gut microbiota‐associated effector immune cells and cytokines can migrate from the GIT to distal sites and provide a tumorigenesis‐permissive environment.^[^ ^37^, ^61^ ^]^

The structurally intact gastrointestinal epithelium barrier consisting of a mucus layer and tightly connected epithelial cells provides the first solid barrier against pathogenic invasion, followed by defensins and cytokines secreted by epithelium cells.^[^ ^24^ ^]^ *H. pylori* induces chronic infections. Adhesion, colonization, and invasion by this species is a risk for gastric cancer. Vacuolating toxin (VacA) secreted by *H. pylori* is a pore‐forming toxin, which can impair the epithelium.^[^ ^62^ ^]^ Oncoprotein cytotoxin‐associated gene A (CagA) damages barrier functions by altering partitioning‐defective polarity complex and depolarizing epithelial cells.^[^ ^63^ ^]^ Claudin‐4 disruption is one barrier‐damaging mechanism that is independent of CagA and VacA.^[^ ^64^ ^]^ Besides, Hwang et al. found that *B. fragilis* toxin damaged E‐cadherin and induced IL‐8 secretion in HT29/C1 cells.^[^ ^65^ ^]^ Other bacterial species (e.g., *Enterococcus faecalis* and *Bacteroides* subspecies) also impair epithelial barrier integrity to facilitate pro‐tumoral bacterial invasion and biofilm formation.^[^ ^24^ ^]^

Once the epithelium barrier is disrupted by bacterial adhesion and internalization, microbes translocate from the lumen to lamina propria and stimulate adaptive immune responses, the roles of which are controversial in tumorigenesis. Proinflammatory and tumor‐associated cytokine‐mediated signaling pathways provide a tumorigenesis‐permissive environment, while adaptive immunity is necessary for tumor suppression and pathogen eradication. Proinflammatory T helper 17 (Th17) and T helper 1 (Th1) are most active in GALT. Th17 responses are known to be two sided in anti‐tumor immunity. Effector and regulatory Th17 differentiation are dependent on specific stimuli and tissue environment.^[^ ^66^ ^]^ A study by Tan et al. revealed that segmented filamentous bacteria promote the maturation of CD4^+^ T helper cells toward the Th17 cell moiety that produces IL‐17 and IL‐22, which protects the mucosa from pathogenic infection.^[^ ^67^ ^]^ Similarly, *Bifidobacterium adolescentis*, a candidate for probiotic preparation, also induces Th17 cells differentiation in the murine intestine.^[^ ^68^ ^]^ Wu et al. demonstrated that classical CD4^+^, but not T cell receptor (TCR) *γδ* ^+^ Th17 cells induced by enterotoxigenic *B. fragilis* could boost tumorigenesis in mice.^[^ ^69^ ^]^ Further understanding of the Yin and Yang role played by Th17 is necessary for manipulating microbiota. In addition, gut microbiota have been implicated in regulation of T cell survival in germ‐free (GF) mice.^[^ ^70^ ^]^ Captanin et al. reported that a significant proportion of T cells isolated from *H. pylori* patients with chronic gastritis and gastric adenocarcinoma were activated by the HP1454 lipoprotein from *H. pylori*. *H. pylori* has also been found to induce T cell maturation toward the Th1/Th17 moiety through the TCR *ζ* chain phosphorylation.^[^ ^71^ ^]^ The impact of regulatory T cells (Tregs) on carcinogens has also not been established. Traditionally, Tregs are thought to be suppressive for anti‐tumor immunity. However, since the gastrointestinal lumen is a considerably hostile environment, Tregs are indispensable for protecting the host from excessive immune responses. Round et al. reported that *B. fragilis*, as a significant human commensal, facilitates the differentiation of Foxp3^+^ Tregs and enhances the production of IL‐10, transforming growth factor‐β 2 (TGF‐*β*2), and chemokine receptor 6 that is associated with the migration of Tregs. These effects were shown to alleviate tissue injury in colitis GF mice.^[^ ^72^ ^]^ *Bifidobacterium infantis* enhanced the expression of Foxp3 on Tregs, increased the protective colonic programmed cell death ligand 1 (PD‐L1) as well as programmed cell death 1 (PD‐1) levels and promoted the immunosuppressive cytokine (IL‐10 and TGF‐*β*1) levels in an experimental acute IBD murine model.^[^ ^73^ ^]^ However, VacA from *H. pylori* activates peripheral Tregs. The immunosuppressive trait of Tregs favors persistent gastric colonization and infection by *H. pylori*, and affects systemic immunity.^[^ ^74^ ^]^ Regulatory B cells also inhibit chronic inflammation to maintain intestinal homeostasis. Mishima et al. documented that enteric microbiota in SPF wild‐type (WT) isogenic C57BL/6J mice induced the activation of immunosuppressive IL‐10‐producing regulatory B cells through the TLR2/MyD88/PI3K pathway.^[^ ^75^ ^]^

Dendritic cells (DCs) in GALT act as an intersection in lymphocyte differentiation, and promote tolerance to commensal microorganisms and immune responses against pathogens. Intestinal effector DCs and regulatory DCs subsets induce the differentiation of distinct subsets of lymphocyte cells.^[^ ^76^ ^]^ Uribe‐Herranz et al. stated that Gram‐positive commensal bacteria‐mediated antigen presentation on CD11b^+^ DCs, and the depletion of these bacteria through oral administration of vancomycin promoted major histocompatibility complex (MHC) I‐dependent activation of anti‐tumor CD8^+^ T cells, thus improving the therapeutic outcomes of radiotherapy on tumor‐bearing mice.^[^ ^77^ ^]^ *Bifidobacterium bifidum* acts as a potent inducer of CD25^+^ Foxp3^+^ Tregs, in a regulatory DCs (MHC II^+^ CD11c^+^ CD11b^+^ CD103^+^ colonic lamina propria DCs) TLR2‐dependent manner. Cell surface β‐glucan/galactan induces mucosal tolerance to a broad range of TCR antigens.^[^ ^78^ ^]^ Tanoue et al. used a mixture of 11 strains from human caecal microbiota to induce IFN‐*γ* ^+^ CD8^+^ T cells. They confirmed that this effect relied on CD103^+^ lamina propria DCs and MHC I a rather than on innate immune pathways.^[^ ^79^ ^]^

Macrophages are important modulators of the activation of various lymphoid cells. The role of tumor‐associated macrophages (TAMs) in shaping a tumor‐permissive microenvironment has not been conclusively defined. Studies have documented that microbiota mediate M1/M2 profile or the homeostasis of macrophages in the lamina propria.^[^ ^80^ ^]^ Scott et al. found that antibiotic administration enhanced the susceptibility of macrophages to bacterial antigens and promoted excessive inflammation. Antibiotic treatment increased the proportion of Ly6C^hi^ MHC class II^+^ immature/intermediate macrophages that had been rapidly derived from monocytes, leading to an increase in IFN‐*γ*‐producing CD4^+^ T cells and Th1 cells.^[^ ^80^ ^]^ Liu et al. found that *F. nucleatum* accelerated M1 macrophage polarization and aggravated colitis.^[^ ^81^ ^]^ The M2 macrophage is a kind of tumor‐infiltrating immune cell, known for its tumor‐promoting ability. Chen et al. reported that *F. nucleatum* diminished anti‐tumor immunity by macrophage polarization toward a M2 phenotype.^[^ ^82^ ^]^ However, Wu et al. found that autoinducer‐2 from *F. nucleatum*, a quorum sensing system molecule mediating communication among species, could boost proinflammatory M1 polarization in vitro, which was beneficial for anti‐tumor immunity.^[^ ^83^ ^]^ The contrasting results show the importance of further research on the role of *F. nucleatum* under different conditions.

### 2.2 Regulating Cell Life Cycle and Epithelial–Mesenchymal Transition (EMT)

Multiple genes for cell cycle regulation control the proliferation and death of cells through several crucial signaling pathways.^[^ ^84^ ^]^ Once mutations of these gene accumulate, neoplasms emerge, and subsequently enhance the risk of cancer development. As a digestive system cancer, CRC is correlated with commensal bacteria for the interplay between them in regard to cell signal pathway modulation.^[^ ^37b^ ^]^

Several genetic alterations (e.g., activation of KRAS, inactivation of p53 and adenomatous polyposis coli [APC]) occur in CRC, among which APC mutation is the most common.^[^ ^84^ ^]^ *β*‐catenin is a major mediator of the APC/Wnt signaling pathway. The APC tumor suppressive gene exerts inhibitory effects on the Wnt pathway‐associated abnormal proliferation by *β*‐catenin degradation.^[^ ^84^ ^]^ Rubinstein et al. found that FadA (a virulence factor from *F. nucleatum*) induced E‐cadherin phosphorylation and activated *β*‐catenin signaling in several kinds of human colon cells. Moreover, the FadA inhibitory peptide negatively regulated the contingent proinflammatory and oncogenic effects of *F. nucleatum*.^[^ ^85^ ^]^ Rubinstein and her co‐workers found that *F. nucleatum* significantly promoted cell growth in HCT116 and LoVo cells after CRC stimulation. They reported that in the process of FadA‐induced *β*‐catenin signaling modulation, Annexin A1, which was selectively expressed in cancerous cells, acted as a linchpin.^[^ ^86^ ^]^ Lu et al. found that *Salmonella* AvrA stabilized *β*‐catenin, thereby enhancing Myc and cyclin D1 expression as well as promoting CRC development and progression.^[^ ^87^ ^]^ Another study found that in contrast to Wnt2 and Wnt11, Wnt1 was downregulated in *Salmonella*‐infected colon cancer mice which diminished inflammatory responses against colitis‐associated cancer.^[^ ^88^ ^]^ With regard to probiotics, their beneficial effects in modulating *β*‐catenin‐associated pathways can be exploited to treat CRC. Ali et al. found that a combination of *Lactobacillus casei* (*L. casei*) and prebiotic inulin exhibited more efficacy against 1,2‐dimethylhydrazine‐induced colon cancer in experimental mice by upregulating phosphorylated JNK‐1, subsequently reducing *β*‐catenin.^[^ ^89^ ^]^

NF‐*κ*B is a ubiquitous transcription factor that is stimulated by various factors including microbes, cytokines, peptide hormones, and DNA damage. Its correlation with cell proliferation, differentiation, and migration indicates its importance in carcinogenesis.^[^ ^90^ ^]^ Yang et al. reported that *F. nucleatum* infection significantly promoted the growth of HCT116 and LoVo cells, as well as tumorigenicity in Apc^Min/+^ Mouse. This was attributed to the upregulation of miRNA21 by *F. nucleatum*‐activated upstream TLR4/MyD88/NF‐kB signal pathway. *F. nucleatum* burden and high expression of miRNA21 were also detected in advanced human CRC tissues with worse prognosis.^[^ ^91^ ^]^ Compared to *F. nucleatum*, the role of *Peptostreptococcus anaerobius* (*P. anaerobius*) in CRC development and progression is much less investigated. Long et al. documented that *P. anaerobius* targeted colonic cancer cells overexpressing integrin *α*_2_/*β*_1_ and stimulated the PI3K‐Akt‐NF‐*κ*B signal pathway, leading to the upregulation of myeloid‐derived suppressor cells (MDSCs) and TAMs as well as to the promotion of colorectal tumorigenesis in Apc^Min/+^ mice.^[^ ^92^ ^]^ On the contrary, probiotic *Leuconostoc mesenteroides* induces tumor cell apoptosis and anti‐inflammatory effects by inhibiting the NF‐kB/AKT/PTEN pathway.^[^ ^93^ ^]^ Furthermore, the cell life cycle is modulated by complex signal pathways that can be regulated by bacterial interventions. For instance, Notch signaling, which promotes secretory cell determination is inhibited by microbiota in zebrafish intestinal epithelium.^[^ ^94^ ^]^ The interaction between microbiota metabolites and mammalian target of rapamycin (mTOR) signaling also plays a role in various cancers.^[^ ^95^ ^]^

Potential pro‐tumoral bacteria also interfere with cell life cycle through host cell DNA damage or epigenetic modulation by various genotoxins.^[^ ^96^ ^]^ Cytolethal distending toxin (CDT) produced by Gram‐negative bacteria presents DNase activity to induce DNA damage and aggravated the carcinogenesis effect of APC.^[^ ^97^ ^]^ He et al. reported that *Campylobacter jejuni* (*C. jejuni*)‐mediated intestinal carcinogenesis in GF Apc^Min/+^ mice through the effect of cdtB (a subunit of CDT) and by exacerbating host DNA damage. Inhibitor of mTOR signal, rapamycin, suppresses this effect of *C. jejuni*, which could be a prevention approach.^[^ ^98^ ^]^ Colibactin is another important genotoxic product of pathobionts. Increased polyketide synthase (pks) genotoxic island (genes of colibactin) from *Escherichia coli* (E. coli) is associated with tumorigenesis in patients with familial adenomatous polyposis.^[^ ^99^ ^]^ Colibactin‐associated genetic lesion is the consequence of several different effects including DNA alkylation through covalent modification and double‐strand breaks.^[^ ^100^ ^]^ Cougnoux et al. found that pks^+^ *E. coli* promoted tumor growth by inducing cellular senescence followed by increasing growth factors.^[^ ^101^ ^]^ Pleguezuelos‐Manzano et al. utilized human intestinal organoids to define pks^+^ *E. coli*‐induced mutational signature, which was also found in CRC cohorts.^[^ ^102^ ^]^

EMT refers to a comprehensive biological process, in which regular arranged epithelial cells in monolayer cultures transform into more‐mesenchymal cells with enhanced plasticity, mobility, and aggressiveness.^[^ ^103^ ^]^ The involvement of EMT in tumorigenesis and metastasis is a health concern.^[^ ^103^, ^104^ ^]^ The impact of microbiota on epithelium barrier functions, immune homeostasis, and cell life cycle regulation through interference in a myriad of signal pathways build the bridge between EMT and cancer pathogenesis.^[^ ^103^, ^104^, ^105^ ^]^ ASPP2 is an inhibitor of EMT.^[^ ^106^ ^]^ CagA from *H. pylori* abrogated barrier functions by altering partitioning‐defective polarity complex and depolarizing epithelial cells through multiple mechanisms including blockade of ASPP2. As a result, CagA‐ASPP2 interaction‐induced predisposition to EMT.^[^ ^63^ ^]^ CagA can also mediate EMT progression through binding GSK‐3*β*, leading to the depletion of GSK‐3 and elevation of Snail protein.^[^ ^107^ ^]^ Wan et al. reported that increased production of IL‐6 and TNF‐*α* by macrophages contributed to EMT process and exerted pro‐tumor effects in antibiotic‐induced intestinal dysbiotic mice.^[^ ^108^ ^]^ Sodium butyrate, a common bacterial metabolite from dietary fibers disintegration in colon, has been shown to inhibit histone deacetylase 4 (HDAC4), thus suppressing TGF‐*β*1‐induced EMT in human hepatoma cells. Sodium butyrate also increased cell cycle blocking protein levels (P27 and P21), as well as anti‐apoptotic protein levels (Bcl‐2 and Bcl‐xL).^[^ ^109^ ^]^ As a result, the migration and invasion of hepatocellular carcinoma (HCC) cells was inhibited by sodium butyrate. To some extent, the result of this study could be correlated to the radiation‐induced intestinal EMT suppression activity of soluble dietary fibers.^[^ ^110^ ^]^ In a retrospective cohort study involving CRC patients of stage iii/iv by Yan et al., enrichment of *F. nucleatum*, which is associated with significantly low E‐cadherin and high N‐cadherin (EMT markers) as well as high Nanog (cancer stem cell markers) levels, was an adverse factor in cancer‐specific survival and disease‐free survival.^[^ ^111^ ^]^ Previous studies on the role of *F. nucleatum* in regulating E‐cadherin elucidate the possible mechanisms.^[^ ^85^, ^86^, ^91^ ^]^

In conclusion, pathobionts are able to intervene cell life cycle and EMT at protein, epigenetic, and genetic levels, while the complicated interplay between various signal pathways are still unclear. However, several potential therapeutic targets have manifested their prospective efficacy in clinical anti‐tumor treatment.

### 2.3 Regulating the Metabolism in the Gut

Metabolic deregulation has been established to be the pathogeny of chronic inflammation and tumorigenesis. It is universally acknowledged that gut microbiota, serving as a special organ, plays an essential role in host metabolism. Exposed to a wealth of nutrients, xenobiotics, and a host of primary metabolites in the digestive tract, gut microbiota participate in the process of degradation, fermentation, absorption of nutrients, and production of bioactive substances, especially in the large intestines.

Bacterial fermentation of dietary fibers and resistant starch is the main mechanism of producing short chain fatty acids (SCFA), which refer to organic fatty acids consisting of 1–6 carbon atoms. Acetic acid, propionic acid, and butyric acid consist of over 90% SCFA in the colon.^[^ ^112^ ^]^ As the most notable type of colonic SCFA, butyrate regulates energy metabolism, protects epithelial barrier, inhibits pathogenic immunological responses, and suppresses tumors.^[^ ^112^, ^113^ ^]^ In human colon, normal cells obtain their energy from SCFA rather than glucose.^[^ ^113^ ^]^ Compared to normal cells, cancer cells are characterized by a shift in major energy sources from oxidative phosphorylation to aerobic glycolysis, termed “the Warburg effect.”^[^ ^114^ ^]^ This phenomenon in cancer cells explains the distinct roles of SCFA in different cells. Instead of being consumed in tricarboxylic acid cycle to provide energy, butyrate acts as an HDAC inhibitor to regulate cell proliferation, differentiation, and apoptosis on an epigenetic level.^[^ ^113^, ^115^ ^]^ Dietary fibers ameliorate carcinogen lesions by increasing protective SCFA levels.^[^ ^116^ ^]^ Donohoe et al. reported that the transplantation of bacterium‐produced butyrate inhibited CRC development in gnotobiotic mice fed with high‐fiber diet by promoting histone acetylation.^[^ ^116^ ^]^ miRNA also mediates butyrate's anti‐tumor effects. Hu et al. found that butyrate deactivated the intronic C13orf25 (oncogenic miR‐17‐92a cluster) promoter, thus leading to p57 activation, which contributed to the anti‐proliferative and pro‐apoptotic effect of butyrate.^[^ ^117^ ^]^ With regard to the immune modulation role of butyrate, Zimmerman et al. reported that butyrate promoted Fas promoter hyperacetylation in T cells, giving rise to Fas‐mediated apoptosis and inflammation suppression. Inducible nitric oxide synthase (iNOS) production was also inhibited by butyrate.^[^ ^118^ ^]^ Furusawa et al. revealed that butyrate from commensal bacteria induces epigenetic upregulation of the Foxp3 gene in CD4^+^ T cells and differentiation of Tregs, which contributes to colonic immunological homeostasis.^[^ ^119^ ^]^ In a study involving the colonic lumen of IBD mice, the depletion of SCFA by antibiotics was detrimental for proinflammatory hypersensitive macrophages‐mediated Th1 responses, which are necessary for defenses against infections.^[^ ^80^ ^]^ It has also been found that butyrate upregulates TLR4, thus enhancing TNF‐*α* production and improving innate immune responses to tumor cells.^[^ ^120^ ^]^ SCFA can also regulate their receptors, G‐protein coupled receptor (GPR) 43, GPR41, and GPR109, on cell surfaces. Wu et al. found that acetate upregulates intestinal Immunoglobulin A (IgA) production in a GPR43‐dependent fashion. Intestinal IgA maintains immune homeostasis and prevents dysbiosis.^[^ ^121^ ^]^ Butyrate‐producing probiotics, for instance, *Clostridium butyricum* (*C. butyricum*), negatively regulates Wnt/*β*‐catenin signaling, activates GPR43 and GPR109A, and activates cyclin‐dependent kinase inhibitor P21WAF1 in CRC tumor cells.^[^ ^122^ ^]^

Primary bile acid is a natural surfactant that is produced within hepatocytes and released in the gut. High fat diets stimulate bile acid secretion and triggers a series of metabolic and immunological dysfunctions.^[^ ^115b^ ^]^ Abundant commensal bacteria in the colon can convert primary bile acids into a large pool of metabolites that serve as regulators in various physiological activities.^[^ ^123^ ^]^ Through deconjugation and dehydroxylation, bacteria in the gut ecosystem converts primary bile acid to secondary bile acids, which are probable colonic carcinogens.^[^ ^123^, ^124^ ^]^ Cao et al. reported that deoxycholic acid promoted inflammation and intestinal carcinogenesis in Apc^Min/+^ mice, with decreased butyrate‐producing bacteria in the microbial community. Microbiota from deoxycholic acid‐treated Apc^Min/+^ mice could upregulate proinflammatory TNF‐*α* and IL‐23p19 levels, downregulate anti‐cancer IFN‐*γ* levels, switch macrophages toward the M2 phenotype, and activate Wnt/*β*‐catenin pathway in recipient SPF Apc^Min/+^ mice.^[^ ^124^ ^]^ Wan et al. also found that cholic acid was converted to deoxycholic acid in Apc^Min/+^ mice and promoted intestinal carcinogenesis by diminishing SCFA‐producing bacteria while inducing IL‐6/STAT3‐associated inflammation. The intestinal carcinogenesis was suppressed by an antibiotic cocktail.^[^ ^115c^ ^]^ Microbial bile acid metabolites also maintain host immunological homeostasis by controlling the balance between Tregs and Th17. Song et al. reported that various primary or secondary bile acids upregulate ROR*γ* ^+^ Tregs in the gut through the bile acid–vitamin D receptor.^[^ ^125^ ^]^

Mediated by gut microbes in the colon, proteolytic fermentation of undigested proteins generate a variety of metabolites (e.g., ammonia, amines, phenol acid, indoles, p‐cresol).^[^ ^126^ ^]^ The carcinogenetic role of these metabolites is associated with high‐protein diet‐induced disease.^[^ ^127^ ^]^ Ammonia is nocuous for gastrointestinal mucosa, lowers the SCFA and elevates the pH value of colonic environments, thereby predisposing them to tumorigenesis.^[^ ^128^ ^]^ P‐cresol is a methyl phenol that is degraded from tyrosine through microbial actions. P‐cresol in the supernatant of fermentative batch cultures inoculated with feces was shown to exert genotoxic activity on colonic epithelium cell model in a dose‐dependent manner.^[^ ^129^ ^]^

Trimethylamine N‐oxide (TMAO) is another essential toxic agent that is produced after choline is metabolized by gut microbiota. Gut microbial actions enhance tumorigenesis in liver through TMAO production, at least partly.^[^ ^130^ ^]^

In addition, some gut microbes are able to improve anti‐tumor properties of phytochemicals from plant‐based foods. Flavan‐3‐ols is a predominant polyphenol in daily diets, and many of its microbial metabolites such as 4‐hydroxy‐5‐(3,4,5‐trihydroxyphenyl) valeric acid exhibit tumor suppressor activities.^[^ ^131^ ^]^ Several gut microbiota species, such as *Weissella confuse* and *Clostridium perfringens*, were found to increase the cytotoxicity of quercetin in specific cancer cell lines in vitro.^[^ ^132^ ^]^

## 3 Gut Microbiota Modulate Responses to Cancer Therapy

**Figure 2:**
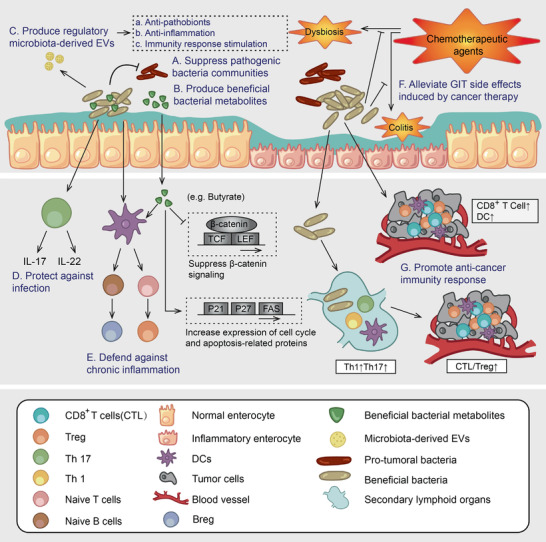
The role played by beneficial bacteria in intestinal homeostasis maintenance and anti‐tumor immunity. A) Suppress the community of pathogenic bacteria. B) Protect the host against bacterial infection. C) Produce regulatory EVs which intervene in the bacterial communities and host physiological activities. D) Produce beneficial metabolites including butyrate and anti‐tumor phytochemical metabolites. E) Maintain immunological homeostasis and inhibit chronic inflammation. F) Alleviate GIT side effects induced by cancer therapy. G) Promote anti‐cancer immunity responses. Specific bacterial communities promote the differentiation of CD8^+^ T cells and enhance sensitivity to cancer therapy. Based on the role played by beneficial bacteria, boosting bacterial communities will favor systemic cancer therapy. Particularly, the microbiota EVs can be potential tools for microbiota modulation. Engineered bacteria based on DDS have natural advantages to be functionalized as cancer therapeutics (e.g., hypoxia tropism, anti‐cancer immune response stimulation).

The roles of microbiota as contingent contributors or suppressors in local and distal oncogenesis has been established. Commensal communities have an impact on systemic anti‐tumor treatment. In correspondence with the systematic metabolic and immunological modulation function of host microbiota, microbial factors influencing chemotherapeutic agent metabolism and anti‐cancer immune responses can determine the outcome of cancer therapy.^[^ ^3^, ^12^, ^133^ ^]^ Furthermore, the severe side effects of chemotherapy and radiotherapy such as gastrointestinal toxicity, partly depend on the function and structure of intestinal commensal bacteria.^[^ ^134^ ^]^ Therefore, for the purpose of rationally modulating human microbiota to optimize the prevention, treatment, and prognosis of malignant tumors, elucidating the mechanisms through which microbial factors affect the outcome of anti‐cancer therapies is extremely important (**Figure** **2**).^[^ ^135^ ^]^

### 3.1 Chemotherapy

Although limited by side effects and non‐selectivity, chemotherapy is still the mainstay of clinical cancer treatment due to its powerful and definite cytotoxicity on cancer cells.^[^ ^133b^ ^]^. Moreover, antibiotics are widely used during chemotherapy to prevent pathogenic infection, which have great impacts on gut microbiota. To improve the efficacy and alleviate the side effects of chemotherapy, microbial factors have raised wide scientific and clinical concern.^[^ ^41^, ^133^, ^135^ ^]^

Specific bacterial species promote resistance to cytotoxic agents by inducing cell autophagy or directly degrading the therapeutic agents. Gut microbiota or intratumoral bacteria play a role in the metabolism of xenobiotics and chemotherapeutic agents. Geller et al. identified intratumoral bacteria from pancreatic ductal adenocarcinoma which could induce resistance of colon cancer cells to gemcitabine. They found that *Gammaproteobacteria* species were the most common in pancreatic ductal adenocarcinoma. These species expressed cytidine deaminase to deactivate gemcitabine, resulting in drug resistance.^[^ ^136^ ^]^ Yu et al. demonstrated that *F. nucleatum* promoted CRC resistance to oxaliplatin (OXA) and 5‐fluorouracil (5‐FU) in vitro by increasing autophagy signaling elements ULK1 and ATG7 expression. Clinical data from colorectal tissue of CRC patients was consistent with this result.^[^ ^137^ ^]^

Moreover, beneficial bacteria enhance the efficacy of chemotherapy by inducing adaptive immune responses and inflammatory processes, resulting in a higher sensitivity to chemotherapy. Viaud et al. found that the administration of cyclophosphamide (CTX) disrupted the intestinal barrier, which facilitated the translocation of Gram‐positive bacterial species (e.g., *Lactobacillus johnsonii* and *Enterococcus hirae*) into mesenteric lymph nodes and spleens in SPF mice. This process induced the differentiation of “pathogenic” Th17 and Th1, which enhanced adaptive immune responses to the tumor.^[^ ^138^ ^]^ A study by Daillère et al. further explained the role of two relevant bacterial species, *E. hirae* and *Barnesiella intestinihominis*, in CTX treatment against MCA205 fibrosarcoma. They reported that *E. hirae*‐induced pathogenic Th17 cells and Th1 cells to increase CD8^+^ T cells and cytotoxic T lymphocyte (CTL)/Tregs ratios, thereby restoring the systemic immune responses impaired by antibiotics. *B. intestinihominis* ameliorate chemotherapeutic efficacy by inducing CD4^+^ Th1 and CD8^+^ CTL cells.^[^ ^139^ ^]^ Kuczma et al. reported that antibiotics significantly reduced CD4^+^ and CD8^+^ T cells producing proinflammatory cytokines (IFN‐*γ* ^+^ TNF‐*α* ^+^), along with CD40L^+^ helper CD4^+^ T cells, thus compromising the efficacy of CTX chemotherapy and facilitating the recurrence of B‐cell lymphoma in mice.^[^ ^140^ ^]^ Bacterial components, such as bacterial ghost (empty envelopes of Gram‐negative bacteria), act as immune‐stimulatory adjuvants in anti‐tumor therapy. Bacterial ghosts improved the therapeutic efficacy of OXA in mice bearing colorectal carcinomatosis, with increased natural killer T (NKT) cells (CD335^+^ CD3^+^), CD8^+^ CD25^+^ T cells, and activated (MHC II^+^) macrophages.^[^ ^141^ ^]^ Immunogenic cell death (ICD) is one of the key mechanisms through which chemotherapy exerts its anti‐cancer effect, dependent on DCs antigen presentation and CTL cell killing. Reactive oxygen species (ROS) production accompanied by DNA damage facilitates the apoptosis of cancer cells and ICD process. Microbiota play a role in activating DCs and inducing CD8^+^ T cells, thus reinforcing the ICD effect.^[^ ^142^ ^]^ Tanoue et al. found that a mixture of 11 strains from human caecal microbiota increased the number of IFN‐*γ* ^+^ CD8^+^ T cells. They proved that this effect relied on CD103^+^ lamina propria DCs and MHC Ia.^[^ ^79^ ^]^ Iida et al. found that an antibiotic cocktail attenuated the induction of ROS‐generating nicotinamide adenine dinucleotide phosphate oxidase 2, thus impairing the efficacy of OXA.^[^ ^143^ ^]^

Microbiota are involved in protecting intestinal barriers and exacerbating the toxic effects of chemotherapy. Irinotecan elicits severe diarrhea and intestinal mucositis, which limits its efficacy in colon cancer therapy.^[^ ^133b^ ^]^ Bacterial enzymes, such as *β*‐glucuronidases, transform the inactive irinotecan metabolite (SN‐38G) to the active metabolite (SN‐38) in GIT, leading to delayed diarrhea.^[^ ^144^ ^]^ Beneficial bacteria supplementation and microbiota restoration attenuate digestive system side effects of chemotherapy through various mechanisms, including maintaining the intestinal mucosa, anti‐inflammation, and reverting dysbiosis. Probiotic *E. coli* Nissle 1917 (EcN) alleviates intestinal barrier dysfunction by enhancing Claudin‐1 expression and inhibiting microbiota dysbiosis.^[^ ^145^ ^]^ According to Chang et al., transplanting microbiota from healthy wild‐type donor mice to mice treated with 5‐FU, leucovorin, and OXA effectively inhibited the TLRs, MyD88, and IL‐6 upregulation as well as intracellular tight junction protein zonula occludens‐1 downregulation caused by chemotherapy.^[^ ^146^ ^]^ Microbiota restoration‐induced Muc‐3 expression and CD11b^+^ myeloid cells accumulation in GIT of cisplatin‐treated mice and facilitated the healing of mucosa.^[^ ^134b^ ^]^ SCFA suppresses intestinal inflammation and protects intestinal barrier functions.^[^ ^112^, ^118^, ^147^ ^]^ Oral administration of Simbioflora (a symbiotic combination of *Bifidobacterium lactis* and several types of *Lactobacillus* plus fructo‐oligosaccharide) was proved to improve the intestinal damage caused by 5‐FU, partly through the production of SCFA.^[^ ^148^ ^]^ Similarly, *Lactobacillus plantarum* (*L. plantarum*) KLDS1.0318 supplementation reduced the production of IL‐2, IL‐4, IL‐6, TNF‐*α*, and IFN‐*γ*, while increased SCFA and lowered pH as well as ammonia concentration, thus improving the outcome of CTX treatment.^[^ ^149^ ^]^ Dysbiosis due to chemotherapy contributes to intestinal dysfunction. Perales‐Puchalt et al. found that intestinal microbiota from untreated mice feces alleviated the increase of Bacteroidaceae and Erysipelotrichaceae family bacteria as well as the decrease of *Ruminococcus gnavus* induced by cisplatin, thus accelerating the restoration of normal ecosystem in the GIT.^[^ ^134b^ ^]^ Recently, a natural product, berberine, was reported to achieve remission of 5‐FU‐induced intestinal mucositis. This effect was attributed to the combination of tight junction restoration, decreasing IL‐1*β*, IL‐6, and TNF‐*α*, increasing butyrate as well as enriching beneficial bacteria.^[^ ^134a^ ^]^

### 3.2 Immunotherapy

The emergence of immunotherapy was a profound development in clinical cancer treatment, as it focuses on the abnormal anti‐cancer immunological functions. In particular, the application of immune checkpoint blockade (ICB) has significantly advanced cancer treatment.^[^ ^150^ ^]^ However, considerable heterogeneity has been shown in response to immunotherapy with resistance and side effects.^[^ ^151^ ^]^ Immunosuppressive tumor microenvironments result in deficient lymphocyte infiltration which suppresses responses to immunotherapy.^[^ ^150^ ^]^ Microbial factors involved in modulating immunological functions have not been fully elucidated. Studies attest to the undeniable role of microbiota in altering the sensitivity of tumors to immunotherapy, which yields a valuable insight into strategies for further optimizing the diagnostic method and treatment outcomes.^[^ ^151^, ^152^ ^]^

Strong correlations have been proven to exist between characteristic commensal bacterial communities and immunotherapeutic efficacy. Microbiota play a role in shaping host immunological functions, from innate immune responses to adaptive immune responses in local and remote lymphoid and myeloid cells. Derosa et al. conducted a cohort analysis and found that antibiotics negatively influenced the efficacy of ICB in patients with advanced renal cell and non‐small‐cell lung cancer (NSCLC).^[^ ^153^ ^]^ Matson et al. found that the commensal microbiota composition, marked by potentially “beneficial” and “non‐beneficial” operational taxonomic units (OTUs) ratio, was significantly associated with the response to anti‐PD‐1 cancer therapy in metastatic melanoma patients. Fecal material from responders strengthened anti‐tumor T cell responses in GF mice, with increased CD8^+^ T cells rather than Foxp3^+^ CD4^+^ Tregs.^[^ ^40^ ^]^ Vétizou found that feces from CTL‐associated antigen‐4 (CTLA‐4) antibody (Ab)‐treated metastatic melanoma patients induced the outgrowth of *B. fragilis* and responses to CTLA‐4 Ab in tumor‐bearing GF mice. By inducing the maturation of intratumoral DCs and IL‐12‐dependent Th1 activation, immunogenic *B. fragilis* restored the efficacy of CTLA blockade in GF mice, which could be abrogated by broad‐spectrum antibiotics.^[^ ^152^ ^]^ Therefore, therapeutic outcomes of CTLA‐4 blockade depend on microbiota composition, especially *Bacteroides* spp.^[^ ^152^ ^]^ Gopalakrishnan et al. found that melanoma patients responding to PD‐1 immunotherapy possessed a higher abundance of bacteria of the Clostridiales order and Ruminococcaceae family as well as lower abundance of Bacteroidales in gut compared to non‐responders. Feces from responders elevated the CD8^+^ T cells as well as CD11b^+^ Ly6G^+^ cells and inhibited suppressive myeloid cells in recipient mice.^[^ ^154^ ^]^ Similarly, Routy et al. found a distinction of dominant commensal species in responders and non‐responders to immune checkpoint inhibitors, among which *Akkermansia muciniphila* enhanced CD4/Foxp3 ratios in tumors and increased C‐C chemokine receptor 9^+^ C‐X‐C chemokine receptor 3^+^ CD4^+^ T cells (a subset of central memory T cells) frequency.^[^ ^155^ ^]^ The diversity of gut microbiota also has impacts on anti‐PD‐1 immunotherapy.^[^ ^154^, ^156^ ^]^ The within‐sample diversity of gut microbiome in anti‐PD‐1 immunotherapy‐responding melanoma patients was significantly higher than in non‐responding counterparts.^[^ ^154^ ^]^ Chinese patients with NSCLC were also investigated by Jin et al. They reported that the alpha diversity of gut microbiome was positively associated with response to PD‐1 Ab Nivolumab, with elevated CD8^+^ T cells and NK cells.^[^ ^156^ ^]^ Immunotherapy is likely to be enhanced by immune responses to ICD, which can be promoted by natural adjuvants from bacteria. For instance, Hancz et al. revealed the role of flagellin as an intensifier of tumor immunotherapy, since it stimulated death receptor‐induced apoptosis of Jurkat T cells.^[^ ^157^ ^]^

In addition to ICB, CpG‐oligonucleotide (CpG‐ODN) immunotherapy partly relies on gut commensal bacteria. The production of TLR‐4‐dependent TNF, a major response to CpG‐ODN, was found to be impaired by the administration of antibiotic cocktails in tumor‐bearing mice. Antibiotics decrease the frequencies of prime innate myeloid cells which produce various proinflammatory cytokines in response to CpG‐ODN.^[^ ^143^ ^]^

The TGF‐*β* inhibitor, Galunisertib, is a promising immunotherapeutic agent for cancer treatment. EcN associates with Galunisertib through increasing the levels of IFN‐*γ* ^+^ CD8^+^ cells and DCs in tumor‐draining lymph modes along with attenuating Foxp3^+^ Tregs and IL‐10 in tumors.^[^ ^158^ ^]^

Broad‐spectrum antibiotics inhibit the immunotherapeutic‐supportive capacity of commensal bacteria.^[^ ^140^, ^143^, ^152^, ^153^ ^]^ For instance, Kuczma et al. found that a broad‐spectrum antibiotic cocktail diminished the efficacy of CD4^+^ T‐cell therapy by inhibiting CTX‐induced microbial translocation into secondary lymphoid organs.^[^ ^138^, ^140^ ^]^ Uribe‐Herranz et al. reported that vancomycin, which targets Gram‐positive bacteria, enhanced the anti‐tumor effects of adoptive T‐cell therapy (ACT) by reinforcing systemic anti‐tumor immunity. Vancomycin treatment ameliorated the tumor suppression efficacy of ACT in Jax mice. The phenotype could be copied by FMT to Har mice. Vancomycin treatment increased the abundance of Bacteroidales S24‐7 family and decreased the abundances of Firmicutes and Clostridiales in gut microbiota of Jax mice. Moreover, tumor‐infiltrating CD8^+^ T cells were significantly increased while reactive T cells, and CD8*α* ^+^ DCs and IL‐12p70 were systemically expanded.^[^ ^159^ ^]^ Also, Vétizou et al. reported that vancomycin improved the therapeutic effects of CTLA‐4 blockade by enhancing the proliferation of Bacteroidales.^[^ ^152^ ^]^ Therefore, a more definite and detailed causality between specific bacterial species and immunotherapy ought to be illuminated in future studies.

Microbiota‐derived EVs refers to outer membrane vesicles (OMVs) of Gram‐negative bacteria and membrane vesicles of Gram‐positive bacteria.^[^ ^160^ ^]^ With membrane encapsulated cytosolic structures and complex enclosed components, microbiota‐derived EVs play a role as messengers in bacteria–bacteria and bacteria–host interactions.^[^ ^47^, ^160^, ^161^ ^]^ Particularly, EVs from probiotics also have the potential to be immunomodulators which suppresses chronic inflammation or acts as adjuvants to cancer immunotherapy.^[^ ^47^, ^162^ ^]^ Furthermore, gut microbiota‐derived EVs are not only located in the GIT, but are also distributed in blood circulation and have access to other organs and tissues.^[^ ^163^ ^]^ With regard to inter‐bacteria intervention, EVs partly mediate quorum sensing, biofilm formation, and cytotoxic effect on other bacterial species.^[^ ^164^ ^]^ For bacteria–host interplay, the most essential effect of EVs is immunoregulation.^[^ ^47^, ^162^ ^]^ Depending on different bacterial species, target cells and bacterial growth status, microbiota‐derived EVs regulate different types of immune responses through distinct signal pathways.^[^ ^47^, ^160^ ^]^ Proinflammatory properties of EVs are largely ascribed to PRRs on cell membranes.^[^ ^47^, ^162^ ^]^ Probiotic *Escherichia* was found to produce OMVs to activate NOD‐1/NF‐*κ*B signaling pathway and to enhance anti‐microbial IL‐6 and IL‐8 expressions.^[^ ^162c^ ^]^ Choi et al. found that probiotic *Lactobacillus paracasei*‐derived EVs suppressed the dextran sulfate sodium‐induced colitis in mice, probably by inhibiting cyclooxygenase‐2, iNOS, and NF‐*κ*B.^[^ ^162b^ ^]^ The positive role of EVs in promoting adaptive immune responses has been confirmed and utilized in vaccine preparation.^[^ ^165^ ^]^ Grandi et al. engineered an OMV‐based cancer vaccine, which enhanced anti‐tumor immune responses due to multiple microbe‐associated molecular patterns and cancer‐specific epitopes on the surface of engineered OMVs.^[^ ^165^ ^]^ In conclusion, beneficial EVs affect immunological modulation in a similar manner to probiotics. Therefore, as components of microecologic systems, EVs are capable of shifting microbiota phenotypes to cancer‐inhibiting ones. In addition, with resistance to enzymes and low pH, EVs are able to deliver various drugs as nanocarriers, which indicates the possibility of combining other cancer therapeutic strategies with EVs.^[^ ^160^, ^163^ ^]^ The use of EVs may also avoid the risk of infections caused by probiotic engraftment in vulnerable individuals.^[^ ^160^ ^]^

### 3.3 Radiotherapy

Radiotherapy is still one of the mainstream treatments for cancers in clinics. Nevertheless, radiation results in great damage on normal rapidly proliferating cells, especially intestinal epithelium, thus, lead to acute enteropathy including nausea, vomiting, abdominal pain, and diarrhea.^[^ ^166^ ^]^ Moreover, delayed radiation enteropathy after radiation therapy can persist for decades and even progress to more severe morbidity.^[^ ^166^, ^167^ ^]^ Therefore, numerous strategies aimed at protecting cancer survivors from great discomforts and health risks associated with radiotherapy have been developed.^[^ ^166^ ^]^ Since microbiota were found to regulate immune responses to malignancy and maintain intestinal homeostasis, cohort studies on its role in modulating radiotherapeutic outcomes have been conducted.^[^ ^3^, ^133^ ^]^

Radiation induces side effects such as dysbiosis and disrupted mucosal homeostasis. Ferreira et al. conducted a cohort study to elucidate the association between gut microbiota and radiation enteropathy caused by pelvic radiotherapy. They found that bacterial diversity was significantly associated with acute radiation enteropathy rather than late enteropathy. A higher, but dynamically decreasing abundance of SFCAs was observed in early enteropathy cohorts. This was accompanied with an increasing abundance of their producers including *Clostridium* IV, *Roseburia*, and *Phascolarctobacterium*, along with a decreasing cytokine level (e.g., IL7, IL‐12, IL‐15, and IL‐16) which regulated microbiota homeostasis.^[^ ^167^ ^]^ The effect of FMT on protecting recipients from radiation‐associated toxicity is a direct proof for the role of microbiota in radiation syndromes. A recent pilot study by Ding et al. established the safety and efficacy of FMT in treating chronic radiation enteritis (CRE). FMT showed satisfactory improvements that was marked by alleviation of diarrhea, rectal hemorrhage, abdominal/rectal pain, and fecal incontinence in three of the five persistent CRE patients. However, the effect was not long lasting. Diversity of gut microbiota was increased in three cases after FMT and the Karnofsky Performance Status was enhanced in all the responders.^[^ ^168^ ^]^ Gerassy‐Vainberg et al. found that pelvic radiotherapy transformed the naive gut microbiota to a more pro‐inflammatory profile which contributed to radiation proctitis in mice. The microbiota from postradiation mice induced TNF‐α and IL‐1β secretion in intestinal epithelial cells in vitro compared with naïve microbiota.^[^ ^169^ ^]^ Another side effect of radiotherapy, intestinal fibrosis, can also be ameliorated by FMT. This is attributed to the HDAC inhibitive activity of SCFAs.^[^ ^109^, ^110^ ^]^ Probiotics *Bacillus licheniformis* has been exploited as a candidate of bacteriotherapy to relieve radiotherapy‐induced gastrointestinal symptoms in pediatric patients. *B. licheniformis* preparation inhibited endotoxin, C‐reactive protein along with TNF‐*α*, IL‐1b, and IL‐6 in serum to alleviate the gastrointestinal inflammatory lesion.^[^ ^170^ ^]^

Through immunological modulation and resistance induction, microbiota plays an indirect role in regulating the efficacy of radiotherapy.^[^ ^133a^ ^]^ The anti‐tumor efficacy of radiotherapy largely depends on the stimulation of antigen presenting cells and induction of cytotoxic lymphocytes in response to radiation‐induced ICD.^[^ ^77^ ^]^ Uribe‐Herranz et al. documented that Gram‐positive commensal bacteria impacted antigen presenting CD11b^+^ DCs, and the depletion of these bacteria by oral vancomycin treatment promoted MHC I‐dependent activation of anti‐tumor CD8^+^ T cells. As a result, the improved therapeutic outcomes of radiotherapy on tumor‐bearing mice had been achieved.^[^ ^77^ ^]^

In conclusion, the relationship between gut microbiota and radiation‐induced side effects is complex and bidirectional, which needs cautious consideration with regard to rational clinical use.

## 4 Modulating Gut Microbiota to Improve Cancer Therapy

Advances in “omics” tools and experimental models have elucidated the role of host–microbiome interplay in health and disease,^[^ ^13^, ^14^, ^18^ ^]^ which have revealed a part of the mechanisms of microbial intervention on cancer therapy.^[^ ^3^, ^9^, ^133^ ^]^ Therefore, modulating microbial communities to improve the outcomes of cancer therapy has become a promising research field.^[^ ^135^ ^]^ Microbiome‐targeted therapy is not a new conception. Since about 1700 years ago in ancient China, FMT had been applied to treat severe diarrhea.^[^ ^171^ ^]^ Besides, traditional approaches to manipulating microbiota include antibiotic administration, pro‐, pre‐, and synbiotics therapy as well as diet modification.^[^ ^37^, ^135^ ^]^ However, these modalities lack selectivity for specific bacterial species or tumor microenvironment‐responsive capacity, which may lead to unexpected effects.^[^ ^38^, ^44^, ^45^ ^]^ Moreover, considering the harsh GIT environment, protection is required for the delivery of active therapeutic agents ^[^ ^45^, ^172^ ^]^. Furthermore, there are drawbacks of the traditional approaches when treating metastasis or tumors in distal sites colonized with TAB.^[^ ^38^ ^]^ Encouragingly, modern material science, engineering, and synthetic biology technology make it possible to establish effective DDS for improving cancer treatment.^[^ ^38^, ^45^ ^]^ Will the gut microbiota become panacea in cancer therapy? To answer the question, in this review, different approaches for modulating the microbiome (**Table** **1**) are compared. After that, seminal works on modern DDS for enhancing the potency of cancer therapy through regulating the gut microbiome or based on engineered bacteria are reviewed. We also have a look into bio‐synthetic technology which can be exploited to find new frontiers of microbiome modulation.

**Table 1:** Comparison of traditional approaches and modern DDS for microbiota manipulation

Microbiota manipulation approaches	Advantages	Disadvantages	Refs.
Fecal microbiota transplantation	Direct introduction of desired microbiota	Ethical and safety issues (e.g., risk of severe infection)	^[^ ^168^, ^171^, ^173^ ^]^
Antibiotics	Control the bacterial infection effectively	Non‐selective for specific bacterial species; risk of dysbiosis	^[^ ^38^, ^174^ ^]^
Simple administration of pro‐, pre‐, and synbiotics	Simple preparation process	Marginal therapeutic effect; failure to effectively modulate intrinsic microbiota	^[^ ^175^ ^]^
Dietary modulation	The mildest approach; compatible with other therapeutics	Only play accessory role	^[^ ^43^, ^127^, ^176^ ^]^
Modern DDS for microbiota modulation	Realize meticulous control on targeting drug delivery and release; maintain the activity of cargos	Complex preparation process; toxicity of carrier material	^[^ ^38^, ^44^, ^45^, ^48^, ^172^, ^177^ ^]^

### 4.1 FMT

As the most direct method of reshaping gut microbiota, FMT had been applied to patients with severe diarrhea in the 4th century, according to the Chinese scientist Ge Hong.^[^ ^171^ ^]^ The development of modern medicine has greatly improved the safety and patient compliance of FMT. Fecal donor selection and screening, fecal matter storage and biobanking, prudent clinical administration and data monitoring are all crucial steps in successful FMT.^[^ ^173a,b^ ^]^ As of yet, the most successful case of FMT application was about recurrent *Clostridium difficile* infection (RCDI).^[^ ^178^ ^]^ The cure rate reached 90% with acceptable side effects.^[^ ^173^, ^178^ ^]^ As a biological therapeutic regimen, FMT was approved by FDA in 2013 for treating RCDI.^[^ ^173a^ ^]^ Subsequently, the executive discretion of FDA removed the restriction from Investigational New Drug on application of FMT to treat RCDI by qualified professionals.^[^ ^173a^ ^]^ The issued guideline for FMT from FDA requests the joint society recommendation to standardize the collection and analysis of fecal materials.^[^ ^173a^ ^]^ Moreover, to reduce the risk of FMT procedures, non‐profit “Netherlands Donor Feces Bank” is recommended as a fine example, for it runs an effective and credible system to optimize the safety of every step of FMT.^[^ ^173b^ ^]^ Not only for RCDI, several studies have provided evidence for the potential of FMT to treat IBD, colitis, and digestive system cancer.^[^ ^42^, ^179^ ^]^ Significant differences in gut microbiota have been observed in healthy and CRC patients based on 16 sRNA and OTU analysis. Generally, *Fusobacterium*, *Escherichia–Shigella*, and *Peptostreptococcusten* are enriched in CRC patients, which can be potential markers for diagnostics.^[^ ^24^, ^115^, ^180^ ^]^ FMT to colitis‐associated colon cancer mice model decreased the tumor size by exerting protective anti‐inflammatory functions through the induction of CD4^+^ CD25^+^ Foxp3^+^ Tregs.^[^ ^179^ ^]^ Furthermore, several seminal studies demonstrated that microbiota from anti‐PD‐1 therapy responders enhanced tumor‐infiltrating T cells and efficacy of immunotherapy.^[^ ^154^, ^155^ ^]^ A clinical trial utilizing FMT to ameliorate the outcome of anti‐PD‐1 therapy on melanoma cancer patients who failed to respond to immunotherapy has started in Israel (NCT03353402). In addition, a pilot study on the possibility of applying FMT on CRE has shown satisfactory outcomes, although large randomized controlled trials are still needed.^[^ ^168^ ^]^ Recently, a project to investigate the safety of FMT for alleviating the toxicity of ipilimumab and nivolumab combination therapy on renal cell carcinoma patients was approved by FDA (NCT04163289). As the application of FMT in cancer therapy is in its infancy, more detailed mechanisms are supposed to be elucidated. The various adverse effects caused by FMT limit its clinical application. The mechanisms of FMT‐related adverse effects and treatment regime have not been established.^[^ ^173^, ^181^ ^]^ Since cancer patients usually suffer from compromised immune functions, stringent donor selection and screening are necessary for protecting patients from severe infections.^[^ ^181^ ^]^

### 4.2 Probiotics

Probiotics refer to a special bacterial group which inhabits mucosa in a moderate amount and is considered as friend of human body health.^[^ ^44^ ^]^ Understanding of the interplay between probiotics and host were initiated by a study on fermented dairy food.^[^ ^172^, ^182^ ^]^ Russian embryologist Elie Metchnikoff first proposed the strategy of improving health by altering colonic bacteria in the early 20th century.^[^ ^182^ ^]^ Based on modern analysis technology, numerous studies have uncovered the potential role of probiotics in modulating metabolic and immunological functions as well as maintaining mucosal homeostasis.^[^ ^44^, ^175^ ^]^ The administration of specific probiotics significantly alleviates bowel toxicity associated with chemotherapy and radiotherapy.^[^ ^44^, ^175^ ^]^ Therefore, probiotics have been explored as non‐invasive therapeutics or protective agents in cancer therapy^.[^ ^175b^ ^]^ *L. plantarum* KLDS1.0318 supplementation improved the outcome of CTX treatment in mice.^[^ ^113^, ^128^, ^149^ ^]^ Related clinical trials will provide more information on the safety and efficacy of probiotics (NCT03704727). To optimize the performance of probiotic preparations, several challenges need overcoming. It is notable that probiotics can induce arresting effects on disease progression under certain circumstances.^[^ ^175^, ^183^ ^]^ Hence, adequate research on the composition of probiotic preparations are required, while whether rigorous preclinical trials are necessary or not will depend on the condition of recipients.^[^ ^175a,c^ ^]^ Sophisticated assessing approaches are the technological basis of probiotics. Qualified Presumption of Safety should concern accurate strain identification, virulence‐associated genes, histological and genetic toxicity, as well as the risk of bacterial translocation.^[^ ^44^, ^175^ ^]^ The resistance gene transfer is also a primary safety concern.^[^ ^175c^ ^]^ In addition, heterogeneous mucosal colonization resistance has been reported in mice models and in healthy human individuals, which limits the colonization of probiotics in GIT and could not be distinguished by fecal configuration.^[^ ^175d^ ^]^ It is, therefore, important to establish the mechanisms through which indigenous gut microbiome exerts resistance to universal probiotics in order to tailor personalized probiotics regimen to overcome the efficacy limitation of microbiome intervention.^[^ ^175d^ ^]^ Shepherd et al. elaborated a metabolic niche favoring the engraftment of specific nutrition‐utilizing *Bacteroides* strain in colonic crypt ecosystem, which could be a potential resolution for probiotic resistance.^[^ ^184^ ^]^

### 4.3 Diet, Prebiotics, and Synbiotics

The opinion that “Let food be thy medicine and medicine be thy food” of Hippocrates is consistent with the theory “homology of medicine and food” in traditional Chinese medicine.^[^ ^37a^ ^]^ In modern society, people have access to better diets with abundant nutrients. The association between diet structure and chronic diseases is a health concern. High‐fiber diets contribute to a low risk of GIT malignancy while high fat and red meat diet favors tumorigenesis.^[^ ^185^ ^]^ Dietary factors play an important role in shaping gut microbial communities and intestinal metabolism, which are associated with CRC risk.^[^ ^43^, ^185^ ^]^ Therefore, modulating gut microbiota by dietary control can be regarded as a moderate modality for preventing CRC.^[^ ^43^, ^127^ ^]^ High‐fat diets significantly decrease microbial abundance, and promotes opportunistic pathogens while suppressing beneficial bacteria.^[^ ^186^ ^]^ Particularly, SCFA‐producing bacteria including *Roseburia* and *Barnesiella* are suppressed by high‐fat diets.^[^ ^186a^ ^]^ Dietary heme from red meat favors a specific alteration of microbial phenotype that is similar to that of dextran sodium sulfate‐induced colitis. It is marked by enrichment in phylum *Proteobacteria*, unc. *Enterobacteriaceae* and *E. coli*.^[^ ^187^ ^]^ As microbiota alteration is the consequence of diet‐related physiological changes including abnormal immune responses, more evidence regarding the direct influence of diet on microbiota is required.^[^ ^54^, ^147^ ^]^

Prebiotic is defined as an indigestible substrate that is utilized by the beneficial microbial communities to promote the growth of colonic bacteria and improve intestinal microecology.^[^ ^43^, ^44^ ^]^ Moreover, the widely accepted oligosaccharides (e.g., fructo‐oligosaccharides, galacto oligosaccharides, xylo oligosaccharides), inulin, polysaccharides, protein and their hydrolysates, as well as natural products from plants have also been proven to have prebiotic effects.^[^ ^45^, ^188^ ^]^ Inulin is one of the most common prebiotics that are used to stimulate the growth of probiotic *Bifidobacterium* in the human colon.^[^ ^189^ ^]^ The phylum *Bifidobacterium* is considered a beneficial due to its positive roles in anti‐pathogen and mucosal homeostasis.^[^ ^68^, ^78^ ^]^ Inulin supplementation restored the ratio of Firmicutes to *Bacteroidetes* that had been upregulated by high‐fat diet and protected the intestines from low‐grade inflammation by increasing IL‐22 production.^[^ ^186b^ ^]^ Fernández et al. redesigned a processed meat product to make it inulin rich, and found that this novel prebiotic food reduced colon polyps in CRC mice, with beneficial alterations in the ratio of Firmicutes to *Bacteroidetes*.^[^ ^190^ ^]^ In addition, a clinical trial showed that inulin and fructo‐oligosaccharide have protective effects on radiation‐induced enteritis.^[^ ^45^, ^192^ ^]^ Polysaccharides from *Ganoderma lucidum* and *Ganoderma sinense* (Chinese herb Lingzhi) significantly increased the amount of tumor‐suppressing *Alistipe shahii* in gut microbiota of mice bearing breast cancer.^[^ ^188^ ^]^ Polyphenols are a broad category of phytochemicals. Polyphenols from berries and their microbial metabolites have been shown to increase the abundance of beneficial *Bifidobacterium*, *Lactobacillus*, and *Akkermansia* in human gut microbiota.^[^ ^191^ ^]^ In addition, synbiotic refers to the combination of prebiotic and probiotics to confer synergistic effects. A combination of *L. casei* and inulin downregulated *β*‐catenin and protected against CRC in mice.^[^ ^89^ ^]^ Improving the delivery efficacy and designing personal prebiotics and dietary supplementation is important for cancer therapy.^[^ ^45^, ^176^ ^]^

## 5 Modulating Gut Microbiota by Drug Delivery Systems to Improve Cancer Therapy

Although the complex functions of human microbiota remain unclear, the known host–microbiota interplay provides directions for the development of cancer therapeutic strategies. Depending on roles in cancer development, commensal bacteria communities can be roughly divided into three different camps: tumor suppressers as friends, cancer drivers as foes, and the neutrals. The first camp refers to beneficial bacteria which inhibit low‐grade inflammation,^[^ ^73^, ^149^ ^]^ protect the mucosal barrier,^[^ ^122^, ^145^ ^]^ produce healthful metabolites such as SCFA,^[^ ^122^ ^]^ and promote anti‐cancer immune responses.^[^ ^79^, ^147^ ^]^ In contrast, the second camp includes invasive bacteria and onco‐microbes, among which *F. nucleatum* and *H. pylori* are typical members.^[^ ^32^, ^193^ ^]^ Obviously, boosting specific beneficial bacterial species and selectively eradicating cancer‐causing microbes are two rational approaches to cancer prevention and suppression. The delivery of growth promoters for probiotics or antibiotic agents requires the navigation to targets on the organ level, the tissue level, and the cell level, with precise control on the release time. Besides, biological active agents need to be delivered to specific sites such as the colon with well‐retained viability.^[^ ^45^ ^]^ Traditional tools are not able to selectively act on the targeted microbiome, which may lead to limited and uncertain therapeutic effects on local or distal malignancy.^[^ ^38^, ^44^, ^45^ ^]^ Furthermore, the translocation of pathogenic bacteria needs to be prevented by delivery strategies.^[^ ^32^, ^38^ ^]^

Compared with freeze‐dry, spray‐dray, electrohydrodynamic processes, and so on, microencapsulation performs better in maintaining the viability of therapeutic agents during the preparation process, protecting probiotics and unstable prebiotics from harsh conditions in GIT and controlling drug release.^[^ ^45^, ^177^, ^194^ ^]^ The most common microencapsulation materials include chitosan, alginate, and gelatin, which endow the formulation with the burst releasing capacity in lower GIT, controlled by both time and bacteria enzymes. Among these materials, some natural polymers such as chitosan also have prebiotic effects.^[^ ^45^, ^172^, ^195^ ^]^

**Figure 3:**
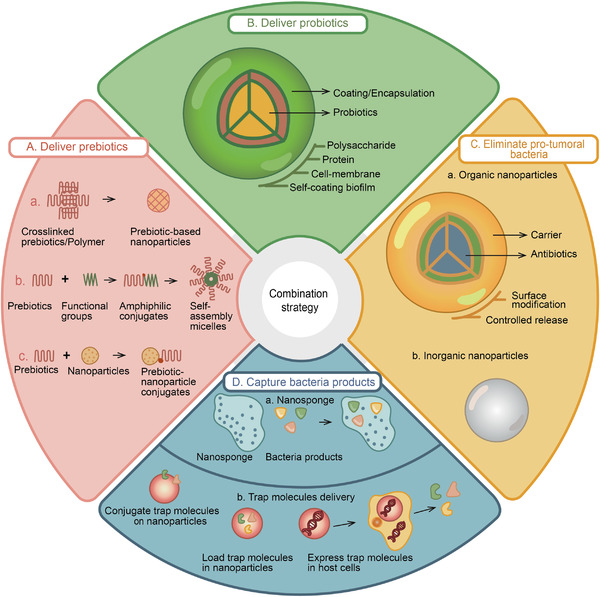
Modern DDS for microbiota modulation to improve the outcomes of cancer therapy. A) Deliver prebiotics. Prebiotics can be used as sustained release materials to prepare nanoparticles for drug delivery, conjugated with functional groups to form self‐assembly micelles, or conjugated to nanoparticles. Prebiotics are appropriate candidates to be delivered with other cancer therapeutic agents to exert synergy effects. B) Deliver probiotics. The encapsulation of probiotics protects their bioactivity from harsh environments. C) Eliminate pro‐tumoral bacteria. Antibiotics delivered by nanosystems are promising to selectively eliminate pro‐tumoral bacteria and avoid dysbiosis, by virtue of nanotechnologies including surface modification, controlled release, and stimuli‐responsive drug release. Inorganic nanoparticles are effective for anti‐infection therapy, overcoming antibiotic resistance of bacteria to some degree. In addition, synergy of pathogen suppression and probiotic promotion based on DDS has shown its promising outlook in cancer therapy. D) Capture bacteria products. Nanoparticles can be designed to bind or adsorb the bacterial products, and release or intracellularly express bacteria products inhibitors.

The nanotechnology‐based DDS for the treatment of both primary and metastatic tumors has enlighten the next revolution in microbiome modulation in local and distal sites.^[^ ^38^, ^196^ ^]^ With the rapid development of nanotechnology such as physical characterization (e.g., size, shape, surface charge), surface modification (e.g., cell membrane coating, functional moiety conjugation, physical adsorption), release control (e.g., stimuli‐responsive release, retained release, multi‐step release) and so on, various kinds of nanomedicines (e.g., liposome, micelle, dendrimer) have been designed as efficient targeting DDS with alleviated toxicity for cancer therapy.^[^ ^196^, ^197^ ^]^ This provides tools for the precise modulation of microbiota to improve the outcome of cancer therapy. Beyond positive targeting delivery of antibiotic by DDS,^[^ ^177^, ^198^ ^]^ more innovative types of nanomedicine regulating commensal bacteria and their products have shown potential in improving cancer therapy (**Figure** **3**, **Table** **2**).

**Table 2:** Recent works on modern drug delivery systems for microbiome modulation to improve cancer therapy

The aim of the work	Drug delivery systems	Key advances	Refs.
Eliminating pro‐tumoral bacteria	Chitosan‐glutamate nanoparticles loaded with amoxicillin (AMX), clarithromycin, and omeprazole	Site‐specific and gastroretentive drug delivery to eradicate *H. pylori*	^[^ ^174^ ^]^
	AMX‐loaded nanoparticles with *H. pylori* UreI channel protein‐targeting moiety on the surface	pH‐sensitive and *H. pylori*‐targeting delivery of antibiotics	^[^ ^198^ ^]^
	Clarithromycin‐loaded gastric epithelial cell membrane‐coated nanoparticles	*H. pylori*‐targeting delivery of antibiotics	^[^ ^48^ ^]^
	*H. pylori* outer membrane‐coated nanoparticles	Inhibiting *H. pylori* adhesion through bacterium‐mimicking approach	^[^ ^203^ ^]^
	Gold nanostars@*H. pylori*‐antibodies nanoprobes	pH and near‐infrared (NIR) dual sensitivity; photoacoustic imaging and eradication of *H. pylori* without disturbing gut microbiome	^[^ ^177d^ ^]^
	M13 phage/Ag nanoparticle complexes	Specific clearance of *F. nucleatum* and activation of the host immune system	^[^ ^209^ ^]^
Boosting probiotics	Tanshinone IIA (TAN)‐loaded nanosized micelles assembled by glutathione‐responsive lipoic acid‐inulin conjugates	Efficient anti‐tumor activity and significantly promoting the growth of *Bifidobacterium longum* *(B. longum)*	^[^ ^172b^ ^]^
	5‐FU‐loaded prebiotics guar gum and xanthan gum nanoparticles	Retarding the release of 5‐FU in upper GIT and protecting the normal colonic bacteria from being damaged by chemical attack of 5‐FU	^[^ ^177a^ ^]^
	Bilberry anthocyanin‐encapsulated low molecular weight citrus pectin (LCP)‐chitosan	Regulating gut microbiome and enhancing the therapeutic effect of *α*‐PD‐L1 mAb on colon cancer	^[^ ^177b^ ^]^
	Fe@Fe_3_O_4_ nanoparticles‐ginsenoside Rg3 conjugates	Remodeling unbalanced gut microbiota and metabolism and suppressing hepatocellular carcinoma development and metastasis	^[^ ^177c^ ^]^
	Alginate‐poly‐l‐lysine‐alginate (APA) membranes encapsulating *Lactobacillus acidophilus* (*L. acidophilus*)	Decreasing overall intestinal inflammation and suppressing intestinal polyp formation in CRC model	^[^ ^194^ ^]^
	Cell membrane‐coated bacteria	Elongating blood circulation, mitigating inflammatory responses, and enhancing tumor accumulation of probiotics	^[^ ^210^ ^]^
Combination strategy for cancer therapy	Prebiotic dextran nanoparticles encapsulating irinotecan linked with phages targeting *F. nucleatum*	Modulating gut microbiota and improving the efficiency of chemotherapy for CRC	^[^ ^46^ ^]^
	Prebiotic dextran‐encapsulated probiotic spores	Boosting SCFA‐producing bacterial communities and inhibiting colon cancer tumor growth	^[^ ^211^ ^]^
Binding bacteria products with nanoparticles	LPS‐trapping nanomedicine containing the plasmid expressing LPS‐binding fusion protein	Relieving the immunosuppressive microenvironment and improving the efficacy of anti‐PD‐L1 mAb for CRC treatment	^[^ ^177f^ ^]^
Engineered bacteria‐based DDS	*ϕ*X174E and anti‐CD47 nanobody‐expressing *E. coli*	Increasing tumor‐antigen‐specific CD8^+^ T cells, rapid regression of A20 tumors, and preventing metastasis	^[^ ^212^ ^]^
	Nanophotosensitizer‐engineered *Salmonella* bacteria with hypoxia‐targeting	High tumor accumulation and effective photothermal effects under NIR laser irradiation	^[^ ^213^ ^]^

1) Biomimetic nanosystem

The biomimetic nanosystem is a broad category referring to those DDS inspired by specific functions of the natural organism. Generally, biomimetic nanosystems are based on either the whole cellular or noncellular organisms, cell membranes or EVs. Biomimetic nanosystems have three major advantages: long circulation time, good biocompatibility, and biological functions inherited from source cells^[^ ^199^ ^]^. For instance, the red cell membrane is the classic material to inhibit rapid clearance of nanoparticles in vivo.^[^ ^200^ ^]^ Moreover, immune cells (e.g., monocytes/macrophages, MDSCs and neutrophils) can be recruited by tumor tissues including the metastases, which is termed as homing capacity.^[^ ^201^ ^]^ Similarly, the hypoxia tropism of anerobic bacteria facilitates their intratumor colonization and proliferation.^[^ ^202^ ^]^ In regard of microbiome intervention, bacterial adhesion to specific host cells is a potential target for precise microbiota modulation. Nanoparticles coated with membrane of bacteria or host cells have been utilized to deliver drugs to certain species or hinder pathogenic adhesion.^[^ ^48^, ^203^ ^]^ Microbiota‐derived EVs, containing cytosol, enzymes, toxin, and gene materials from source bacteria, partly mediate bacteria–bacteria and bacteria–host interactions.^[^ ^160^ ^]^ Therefore, EVs are potential modulators of microbiota and the immunity responses of their host, with the capacity of delivering various drugs.^[^ ^47^, ^160^, ^163^ ^]^ Bacteriophage is a generic term of viruses that infect microorganisms such as bacteria, fungi, algae, actinomycetes, or spirochaetes. As natural enemy to certain bacteria, high‐specific phages enrich the toolkits of biomaterials which are utilized to develop DDS targeting cancer‐causing pathogens.^[^ ^46^ ^]^

2) Combination delivery

Combination delivery refers to the drug delivery strategy that co‐deliver two or more therapeutic agents simultaneously, which can be easily achieved by nanotechnology‐based DDS.^[^ ^204^ ^]^ The synergistic effect of different drugs improve the outcome of chemotherapy.^[^ ^204^ ^]^ Moreover, nano‐devices are favorable for combining cancer therapy and microbiota modulation. Some prebiotics (e.g., chitosan resistant to acid, pectin broken down only by bacteria) are suitable candidates as materials of drug delivery vesicles targeting microbiota‐rich sites in the colon, and they bring synergy effects with probiotics or chemotherapeutic agents.^[^ ^45^, ^172^, ^177^, ^195^, ^205^ ^]^ Co‐delivery of chemotherapeutic agents and pre‐, pro‐, or synbiotics presented the potential in preventing dysbiosis and ameliorating intestinal toxicity.^[^ ^46^, ^172^, ^177^ ^]^ The efficacy of this strategy will be reinforced by using phages with the cancer‐causing bacteria‐eliminating capacity.^[^ ^46^ ^]^.According to different structures of nanocarriers, multiple patterns have been designed to load multiple drugs. Core–shell nanoparticles with hydrophobic drugs in the core and prebiotics in the shell,^[^ ^46^ ^]^ prebiotic‐drug conjugates,^[^ ^177a^ ^]^ as well as drug‐loaded self‐assembly micelles formed by amphiphilic prebiotic derivatives have been reported as combinational therapy platforms.^[^ ^172b^ ^]^

3) Tumor environment‐responsive DDS

The DDS consist of bio‐responsive materials that tend to change their characteristics in the specific microenvironment in tumor sites, where there are varied stimuli factors for drug release, including acidic pH, hypoxia, higher glutathione concentrations, high expression of certain enzymes.^[^ ^197^ ^]^ Besides tumor cells, stromal cells and bacteria in the tumor microenvironment are also sources of stimuli.^[^ ^201^, ^206^ ^]^ For instance, abnormal bacteria communities marked by *Proteobacteria* and *Bacteroidetes* have been found in pancreatic cancer tissues compared to normal ones.^[^ ^206^ ^]^ Therefore, bacterial communities‐responsive nanosystems are promising for tumor‐selective drug release. Wang et al. developed a triple‐layered nanogel which could be degraded by lipase‐secreting bacteria and release its cargo at the bacteria colonization.^[^ ^207^ ^]^ This seminal work demonstrated a novel strategy to control drug release with the aid of the microorganism in the tumor environment.

On the other hand, obligate and facultative anaerobes own natural hypoxia tropism and may have a positive effect on stimulating anti‐tumor immune responses.^[^ ^202^ ^]^ Therefore, engineered microorganisms or EVs from anaerobes can act as targeting drug delivery vesicles or efficient vaccines for cancer therapy.^[^ ^165^, ^202^, ^208^ ^]^

### 5.1 Eliminating Pro‐Tumoral Bacteria by DDS

**Figure 4:**
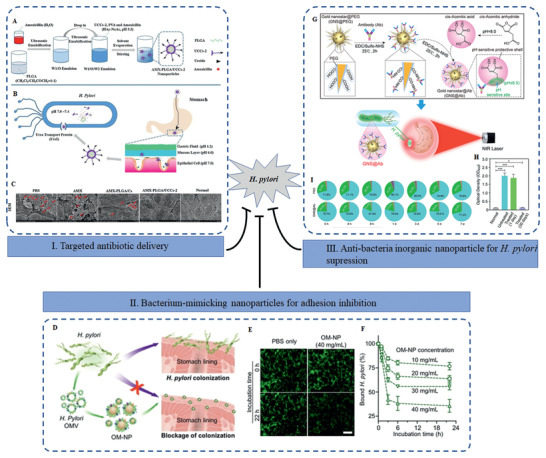
DDS for eliminating pro‐tumoral *H. pylori*. A) Schematic representation of the preparation of AMX‐PLGA/UCCs‐2 nanoparticles. B) The strategy and process to eradicate *H. pylori* using the pH‐sensitive targeting AMX‐PLGA/UCCs‐2 nanoparticles. C) Scanning electron microscopy images of gastric tissue of normal mice or *H. pylori*‐infected mice after treated with PBS, AMX, AMXPLGA/Cs nanoparticles, and AMX‐PLGA/UCCs‐2 nanoparticles. Reproduced with permission.^[^ ^198^ ^]^ Copyright 2018, Elsevier Ltd. D) Schematic representation of OM‐NPs to inhibit *H. pylori* adhesion on the stomach lining. OM‐NPs were prepared by coating polymeric cores made from poly(lactic‐*co*‐glycolic acid) (PLGA) with *H. pylori* outer membranes containing the adhesins that are critical for bacterial colonization. By mimicking the adhesion of *H. pylori* onto gastric epithelium, OM‐NPs occupy the binding sites and hence inhibit the colonization of the bacteria. E) Fluorescence images of *H. pylori* on AGS cells before and after incubation with PBS or OM‐NPs (40 mg  mL^−1^). Green represents FITC‐labeled *H. pylori*. Scale bar = 25 µm. F) Remaining *H. pylori* on AGS cells when incubated with OM‐NPs (10, 20, 30, and 40 mg mL^−1^) at various time points. Error bars represent standard deviations (*n* = 3). Reproduced with permission.^[^ ^203^ ^]^ Copyright 2019, Wiley‐VCH. G) Schematic preparation of pH‐sensitive GNS@Ab and application for targeted imaging and photothermal therapy of *H. pylori* with antibiotic resistance. H) Enzyme‐linked immunosorbent assay for detecting *H. pylori* antigen in stool samples. Data represent mean ± SD (*n* = 5, ^*^ *p* > 0.05, and ^***^ *p* < 0.01). I) The phyla‐level richness of PBS and GNS@Ab groups at different time (0 h, 4 h, 8 h, 1 day, 3 days, 5 days, and 15 days). Reproduced with permission.^[^ ^177d^ ^]^ Copyright 2019, Elsevier Ltd.

Traditional administration of broad‐spectrum antibiotics lacks the selectivity for certain pathogenetic bacteria and may result in health risks such as dysbiosis. Antibiotics‐delivering nanomedicine has been designed for specific eradication of pathogenetic bacteria.^[^ ^38^, ^174^ ^]^ For the treatment against *H. pylori* to prevent gastric carcinoma, the extreme low pH (pH 1–2) is regarded as a stimulus for drug release.^[^ ^32^, ^174^, ^195^ ^]^ Ramteke et al. reported nanoparticles based on gastric fluid‐sensitive chitosan‐glutamic acid conjugates containing triple antibiotic therapeutic agents.^[^ ^174^ ^]^ To target *H. pylori* at the cellular level, more abundant strategies could be exploited. Luo et al. prepared AMX‐loaded poly(lactic‐co‐glycolic acid) (PLGA) nanoparticles with urea‐modified UCCs‐2 on the surface for targeting Urel, a specific urea transport channel protein on *H. pylori* (**Figure** **4**A).^[^ ^198^ ^]^ PLGA was used as a sustained and controlled material to allow burst release of drugs in *H. pylori* (pH 7.4), while to hinder the release in gastric fluid (pH 1.2) and the mucous layer (pH 6.0) (Figure 4B). The nanoparticles presented excellent anti‐*H. pylori* activity and biocompatibility in vivo, entering *H. pylori* via Urel (Figure 4C).

Since the biomimetic nanomedicine presents not only superior biocompatibility and long circulation time, but also the inheritance of biofunctions from source cells or products such as EVs, it has shown outstanding potential in the treatment of orthotopic and metastatic tumors.^[^ ^199^ ^]^ The most commonly used biomaterial is the biological membrane which possesses phospholipids, glycolipid, glycoprotein, and an abundance of receptors and ligands. The coating by biological membranes endows DDS with the capacity of adhesion or tropism to tumor sites.^[^ ^199^ ^]^ The colonization of *H. pylori* in the stomach requires adherence to gastric epithelial cells, which is mediated by the interaction between surface signature of both sides.^[^ ^32^, ^48^, ^63^ ^]^ Therefore, inhibiting the adhesion of pathogenic bacteria to the host cells is a potential antibacterial strategy. Compared with conjugating ligands on nanoparticles, coating with natural cell membrane by the “top‐down” method is a more efficient way to obtain the capacity of adhering to source cells.^[^ ^48^, ^203^ ^]^ Wei et al. reported a biomimetic nanomedicine based on the *H. pylori* outer membrane‐coated PLGA nanoparticle, which acted as a contender of *H. pylori* to adhere to gastric epithelial cell (AGS cell) membranes on a dose‐dependent manner and performed no toxicity (Figure 4D–F).^[^ ^203^ ^]^ From the opposite direction, Angsantikul et al. reported the biomimetic clarithromycin‐loaded PLGA nanoparticles coated with membranes of AGS cells.^[^ ^48^ ^]^ Compared with polyethylene glycol (PEG)‐coated PLGA nanoparticles, AGS membrane‐coated clarithromycin‐loaded PLGA nanoparticles efficiently attached to *H. pylori* and suppressed the *H. pylori* SS1 strain infection in the mice model with undetectable toxicity. The colonization and biofilm formation of *F. nucleatum* are potential risk factors of CRC development.^[^ ^36^ ^]^ The enrichment of *F. nucleatum* in colon adenocarcinomas partly depends on overexpressed Gal‐GalNAc in CRC cells, suggesting that the adhesion of *F. nucleatum* through Gal‐GalNAc can be a target for CRC prevention or treatment.^[^ ^214^ ^]^ Nevertheless, whether the membrane‐coating strategy can be utilized to inhibit pro‐tumoral bacteria adhesion or design the DDS targeting cancer‐causing pathogens other than *H. pylori* has not been stated. Furthermore, the inhibitors or ligands of bacterial adhesion molecules are also candidate cargos for modern DDS.^[^ ^36^, ^214^ ^]^ In another study by Zhi et al., anti‐*H. pylori* polyclonal antibodies were utilized as targeting moieties to prepare a pH‐sensitive theranostic system of *H. pylori* infection based on gold nanostars (GNS@Ab) (Figure 4G).^[^ ^177d^ ^]^ When wrapped in gold nanostars, the flagella rotating and spiral movement of *H. pylori* were severely restrained, thus the reproduction and pathogenic virulence were largely impaired. GNS@Ab selectively bound to *H. pylori* but not other common bacteria (e.g., *E. coli*), and eradicated *H. pylori* without significant cytotoxicity on gastric epithelial cells in vitro. GNS@Ab showed significant targeting capacity to *H. pylori* in stomach (Figure 4H). The NIR laser‐stimulated photothermal effect of gold nanostars resulted in efficient destruction of *H. pylori* in vivo, overcoming the resistance of clarithromycin without significant disturbance on gut microbiome composition and diversity (Figure 4I).

**Figure 5:**
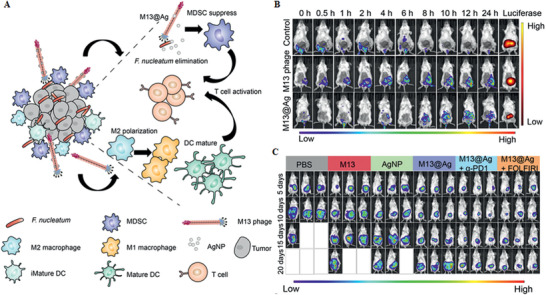
Eliminating pro‐tumoral bacteria with a bioinorganic hybrid system for CRC therapy. A) Schematic illustration of phage‐based bio/abiotic hybrid system (M13@Ag) to regulate gut microbes for cancer‐specific immune therapy. M13@Ag reversed immunosuppressive TME for activation of anti‐tumor immune responses. B) Representative In Vivo Imaging System images presenting the phage accumulation in orthotopic CT26‐luc tumors after treatment with wild phages (without Fn‐binding ability), M13 phages (with Fn affinity), and M13@Ag (n = 3). C) In vivo bioluminescence imaging of orthotopic CT26‐luc tumor‐bearing mice after receiving PBS, M13 phages, AgNP, M13@Ag, and M13@Ag combined with *α*‐PD1 or FOLFIRI [5‐FU (30 mg kg^−1^), leucovorin (90 mg kg^−1^), and IRT (16 mg kg^−1^)] treatments (*n* = 5). Reproduced with permission.^[^ ^209^ ^]^ Copyright 2020, AAAS.

Though these targeting delivery systems for antibiotics have shown potential in gastric adenoma prevention, the information on gastric cancer therapy is still scarce. There have also been reports about exploiting the antibacterial strategy for CRC therapy, with the agents other than antibiotics. To enhance the specificity of antibacterial therapy, Zhang et al. developed a phage‐based bioinorganic hybridization system based on the phase displayed technology.^[^ ^209^ ^]^ This nanosystem consisted of electrostatically assembled *F. nucleatum*‐binding phage strain and silver nanoparticles (**Figure** **5**A). Compare to wild‐type phages, M13 phages showed superior binding capacity to *F. nucleatum*‐enriched tumors and prolonged reservation in tumor site (Figure 5B). As a result, pro‐tumoral *F. nucleatum* was eliminated efficiently and the growth of CRC tumors was significantly suppressed in mice (Figure 5C). The remodeling effects on immunosuppressive tumor microenvironment was demonstrated by improvement of mature DCs, M1 macrophages, and CD8^+^ toxic T cells, as well as depleted MDSCs. Also, IFN production in tumor was upregulated and the amount of tumor‐derived factor interleukin‐10 (IL‐10) changed oppositely.

Moreover, to achieve satisfactory efficiency in modulating gut microbiota for improving cancer therapy, combining more precise antibacterial therapeutics with anti‐cancer agents will be promising.^[^ ^46^ ^]^ To avoid unintended dysbiosis caused by therapies, deep understanding of the host–microbiota and interspecies relationships is required. In addition, bacterial biofilms can be the potential target for DDS considering its role in promoting chronic infections and associated carcinogenesis in GIT.^[^ ^51^, ^53^, ^55^, ^209^ ^]^

### 5.2 Boosting Probiotics for Cancer Therapy with DDS

**Figure 6:**
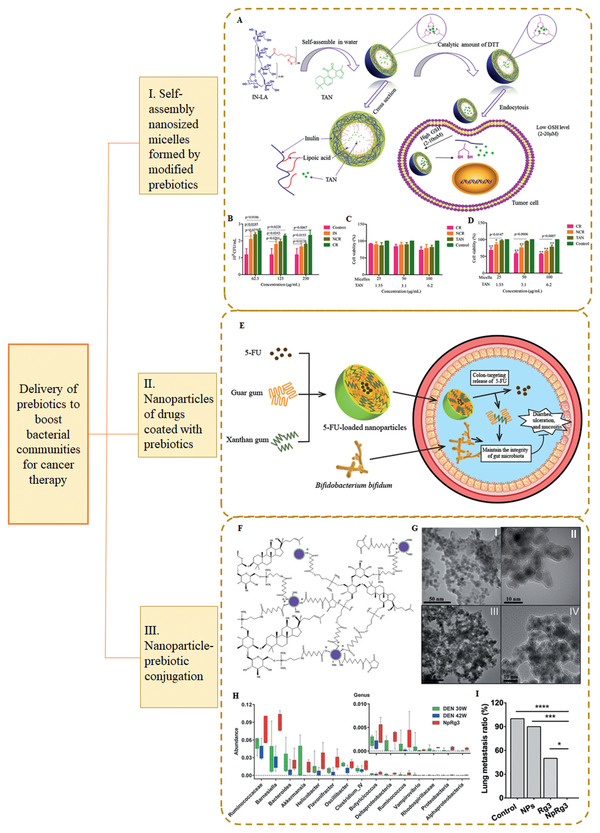
Delivering prebiotics to boost probiotics for cancer therapy. A) Schematic illustration of drug loading and glutathione‐responsive drug release from drug‐loaded cross‐linked (CR) micelle at a high concentration of glutathione in cancer cells. The micelles will eventually be degraded to nontoxic products, inulin, and lipoic acid. B) Effect of IN, TAN‐loaded NCR, and CR micelles on the proliferative activity of *B. longum*. C) Toxicity of TAN‐loaded NCR and CR micelles and free TAN in HT29 cells for 24 h. D) Toxicity of TAN‐loaded NCR and CR micelles and free TAN in HT29 cells for 48 h. Data are presented as mean ± SD. Reproduced with permission.^[^ ^172b^ ^]^ Copyright 2018, Elsevier Ltd. E) Schematic of 5‐FU‐loaded guar gum and xanthan gum nanoparticles. The natural gums play a dual role of achieving colon‐targeted release of 5‐FU and maintain the integrity of gut microbiota along with probiotics supplementation.^[^ ^177a^ ^]^ F) The molecular structure of NpRg3. Fe@Fe_3_O_4_ nanoparticles were synthesized with a metal salt reduction and redox process via a programmed microfluidic process, and conjugated with ginsenoside Rg3 by our invented coupling process sequence. G) The ultrastructures of Fe@Fe_3_O_4_ and NpRg3 were observed by transmission electron microscopy. The apparent core–shell structures of Fe@Fe_3_O_4_ (I and II) and NpRg3 (III and IV) were shown, and NpRg3 had significant organic coupling compared to that of Fe@Fe_3_O_4_. H) Compared with the DEN‐30W group, 15 genera were decreased in the DEN‐42W group, but increased after NpRg3 application. I) The ratio of HCC metastasis to the lung among the four groups. ^*^ *p* < 0.05, ^***^ *p* < 0.001, and ^****^ *p* < 0.0001. Reproduced with permission.^[^ ^177c^ ^]^ Copyright 2019, Wiley‐VCH.

*A. muciniphila* and *Bifidobacterium* have been found to reinforce the efficacy of chemotherapy and immunotherapy due to their roles in regulating systemic immune responses.^[^ ^147^, ^155^ ^]^ In addition, gastrointestinal toxicity of chemotherapeutic agents can be mitigated by beneficial bacteria or microbiota restoration.^[^ ^134^, ^145^, ^146^, ^148^, ^149^ ^]^ Promoting the proliferation of probiotics will help to transform commensals to a cancer therapy favorable phenotype. Biostimulation, a concept in ecology, refers to nourishing the indigenous microorganisms in soil to promote decontamination.^[^ ^172a^ ^]^ Similarly, prebiotics have been widely utilized as fertilizers to boost beneficial bacteria which improves the outcome of cancer therapy in “mucosa soil”.^[^ ^44^, ^175^ ^]^ The differences in the gut microbiota composition among individuals have been observed in many studies,^[^ ^2^, ^10^ ^]^ and prebiotics possess innate selectivity for probiotics which degrade and metabolize prebiotics as nutrients,^[^ ^189^ ^]^ which are more rational therapy tools than those targeting certain species.^[^ ^195^ ^]^ The indigestibility of the most common prebiotics, such as inulin and oligo‐saccharides, is favorable for the delivery to the colon.^[^ ^45^ ^]^ Inulin is a classic growth promoter for *B. longum*, which is generally regarded as a positive modulator of intestinal functions and suppresses the progression of CRC as well as improves the systemic cancer immunotherapy through activating intratumoral CD8^+^ T cells.^[^ ^175b,d^ ^]^ Wang et al. established an inulin‐based glutathione‐responsive DDS with anti‐tumor and probiotic effects (**Figure** **6**A–D).^[^ ^172b^ ^]^ Lipoic acid and inulin were crosslinked via thiol‐disulfide bonds to synthesize amphiphilic copolymers which self‐assembled into nanosized micelles, with TAN, an anti‐cancer and anti‐inflammatory agent encapsulated (Figure 6A). The TAN‐loaded micelles showed the efficient anti‐proliferation activity on HT‐29 cancer cells and significantly promoted the growth of *B. longum*. By virtue of surface modulation and the pro‐drug strategy, this work provided a promising prospect on targeting and combined delivery of prebiotic and chemotherapy or immunotherapy agents through prebiotic‐based DDS to display synergistic effects (Figure 6B–D). Singh et al. developed an oral colon‐targeting nanomedicine based on prebiotics (xanthan gum and guar gum) and 5‐FU, which was administrated along with a probiotic *B. bifidum*.^[^ ^177a^ ^]^ High molecular weight xanthan gum and guar gum had advantages including retarding the release of 5‐FU in upper GIT compared to free 5‐FU and protecting the normal colonic bacteria from being damaged by chemical attack of 5‐FU (Figure 6E). The synergy of prebiotics and probiotic *B. bifidum* further enhanced this effect, and effectively ameliorated the diarrhea and colonic histopathological changes caused by the chemical attack.^[^ ^145^ ^]^ These studies indicated that prebiotics applied as vehicles for anti‐cancer agents delivery had potential to promote the colon cancer chemotherapy, though trials on their application in human bodies are still scarce.

Besides carbohydrates, some phytochemicals such as flavonoids also promote the growth of beneficial bacteria.^[^ ^127^ ^]^ For instance, anthocyanins have been proven to shift the gut microbiota to a healthier phenotype containing enriched beneficial bacteria with a higher diversity, which indicates potential favorable effect on cancer therapy.^[^ ^155^, ^215^ ^]^ However, poor digestive stability and oral bioavailability limit the effectiveness of these chemicals in modulating gut microbiomes.^[^ ^45^, ^215^ ^]^ Modern delivery technology has been employed to overcome these hurdles. Liu et al. encapsulated bilberry anthocyanins by chitosan and LCP, as a gastrointestinal digestion‐resistant combo for microbiota modulation^[^ ^177b^ ^]^ The encapsulation significantly protected anthocyanins from simulated intestinal fluid. The anthocyanin combo tended to restore the alpha diversity of gut microbiota and the Firmicutes to *Bacteroidetes* ratio which were decreased by *α*‐PD‐L1/LCP‐chitosan administration. The result demonstrated synergy effects between anthocyanins and LCP‐chitosan. Bilberry anthocyanins promoted the production of SCFA and intratumoral CD4^+^ T cell infiltration. As a result, anthocyanins effectively enhanced the therapeutic efficacy of the *α*‐PD‐L1 mAb in mice bearing MC 38 colon tumors and this effect was intensified by the encapsulation with LCP‐chitosan.

In another nanosystem, ginsenoside Rg3 acted as both an anti‐cancer agent and a gut microbiota modulator.^[^ ^177c^ ^]^ Ren et al. tested the microbiota‐modulating and anti‐tumor effects of a liver‐targeting nanomedicine based on the conjugate of Fe@Fe_3_O_4_ nanoparticles and ginsenoside Rg3 (NpRg3) (Figure 6F,G).^[^ ^177c^ ^]^ The gut–liver axis played an important role in HCC development, and gut microbiota built a bridge between distinct organs.^[^ ^31^ ^]^ In the dimethylnitrosamine (DEN)‐induced orthotopic HCC mice model, 15 genera in gut microbiota including Ruminococcaceae, *Bacteroides*, and *Akkermansia* were decreased and the metabonomics presented a tumor progression profile. The changes of the gut microbiota composition and gene functions caused by HCC including the increase of tumor‐promoting chemokines, such as LPS‐induced CXC chemokines, and the decrease of tumor‐inhibiting chemokines including IFN‐*γ*, IL‐1*β*, and IL‐12. But NpRg3 relieved the detrimental change of gut microbiota composition (Figure 6H) and slowed the tumor progression and lung metastasis of HCC (Figure 6I). This study indicated a new modality for improving HCC treatment by modulating the gut microbiota.^[^ ^31^, ^172^ ^]^

**Figure 7:**
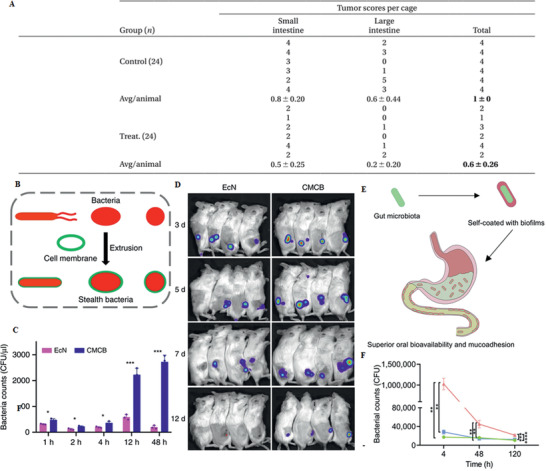
Delivering probiotics for microbiota modulation. A) The efficacy of the microencapsulated live *L. acidophilus* cells on animal tumorigenesis using histochemical analysis. The number of tumors in Apc^Min/+^ mice was scored according to 0–5 scale. Control (*n* = 24) animals were gavaged 0.3 mL of 0.85% saline solution and treatment (*n* = 24) animals were gavaged with APA microencapsulated *L. acidophilus* bacterial cells blended in 2% M.F. yogurt. Reproduced with permission.^[^ ^194^ ^]^ Copyright 2016, Taylor & Francis. B) Schematic illustration for the preparation of CMCB by extruding bacteria with cell membranes. C) In vivo blood reservation of bacteria. EcN or CMCB (1 × 10^7^ CFUs) were injected through the tail vein and blood was withdrawn intraorbitally at the indicated time points, diluted and spread onto LB agar plates. Plates were incubated at 37 °C for 24 h prior to enumeration. Significance was assessed using Student's *t*‐test, giving *p*‐values, ^*^ *p* < 0.05, ^**^ *p* < 0.01, ^***^ *p* < 0.005. D) Tumor imaging of 4T1 tumor‐bearing mice at 3, 5, 7, and 12 days post‐injection of EcN or CMCB expressing LuxCDABE (1 × 10^7^ CFUs). Reproduced with permission.^[^ ^210^ ^]^ Copyright 2019, Springer Nature. E) Schematic illustration of bioinspired oral delivery of gut microbiota with superior oral bioavailability and mucoadhesion by self‐coating with biofilms. F) Total amounts of *Bacillus subtilis* (BS), biofilm‐coated BS, and biofilm fragments‐coated BS retained in the GI tract. Error bars represent SD (*n* = 5). ^*^ *p* < 0.05, ^**^ *p* < 0.01, ^***^ *p* < 0.001, ^****^ *p* < 0.0001, Student's *t*‐test. Reproduced with permission.^[^ ^216^ ^]^ Copyright 2020, AAAS.

Compared with prebiotics therapy, delivering probiotic is a more direct strategy to improve cancer therapy through microbiomes. Various technologies (e.g., spray drying, freeze‐drying, electrohydrodynamic processes, and fluidized bed drying) have been utilized to protect probiotics from the GIT environment.^[^ ^45^, ^194^, ^205^ ^]^ However, these strategies fail to control the release of probiotics. The delivery of probiotics to colon benefits a lot from the microencapsulation technology, which protects the probiotics in harsh GIT conditions such as high acidity in stomach, immune clearance, bile acid, and digestive enzymes, allows the competition of probiotics with commensal microflora, and ensures the time‐controlled or stimuli‐responsive release of probiotics in certain sites such as the colon.^[^ ^45^, ^194^ ^]^ As a widely characterized probiotic, EcN played a positive role in defending against the invasion of pathogens. *C. jejuni*‐mediated intestinal carcinogenesis in GF Apc^Min/+^ mice.^[^ ^98^ ^]^ EcN inhibited the invasion of *C. jejuni* in HT‐29 cells via enhancing the intestinal barrier function.^[^ ^177e^ ^]^ EcN also alleviated the intestinal barrier dysfunction by retaining Claudin‐1 expression and inhibiting microbiota dysbiosis.^[^ ^145^ ^]^ Mawad et al. reported that the encapsulation by alginate and chitosan efficiently maintained the viability of EcN in simulated gastric and intestinal fluid, as well as enhanced the inhibition effect of EcN on *C. jejuni* adhesion with intestinal cells compared to free EcN.^[^ ^177e^ ^]^ Microcapsules also benefited the targeting delivery of probiotics like phylum *Bifidobacterium* and *Lactobacillus*. For instance, Prakasha et al. reported that *L. acidophilus* in APA microencapsules suppressed the formation of polyp during intestinal carcinogenesis in the APC^Min/+^ model (**Figure** **7**A).^[^ ^194^ ^]^

Besides commonly used carriers for probiotic delivery such as polysaccharides and proteins, novel approaches have been proposed to encapsulate probiotics for biomedical applications. Liu and his colleagues prepared a cell membrane‐coated EcN system as a stealth probiotic (Figure 7B).^[^ ^210^ ^]^ Cell membrane‐coated bacteria (CMCB) were proved to induce a low inflammatory response and displayed longer blood circulation and well‐protected inherent bioactivities (Figure 7C). More importantly, the coated bacteria exhibited significantly enhanced colonization ability in tumors compared to uncoated ones (Figure 7D), which indicated the potential of this bacteria delivery system in biomedical applications. Recently, they reported another platform for the oral delivery of gut microbiota.^[^ ^216^ ^]^ Inspired by biofilms produced by bacteria to combat survival threats, they prepared the *Bacillus subtilis* self‐coated with biofilms mainly consisting of the exopolysaccharide and proteins, which exhibited obviously higher GIT tolerance and the mucoadhesion capacity (Figure 7E). As a result, the oral bioavailability was improved (Figure 7F). Although the therapeutic efficacy of these engineered bacteria on specific diseases was not studied, it provided a new idea for designing probiotic delivery systems.

### 5.3 Combination Strategy Based on DDS

The capacity of co‐delivering various therapeutic agents for combined therapy is one of the most attractive merits of nanosystem.^[^ ^38^ ^]^ We highlight the works applying the integrated gut microbiota modulation strategy from Zhang's group in this part.

**Figure 8:**
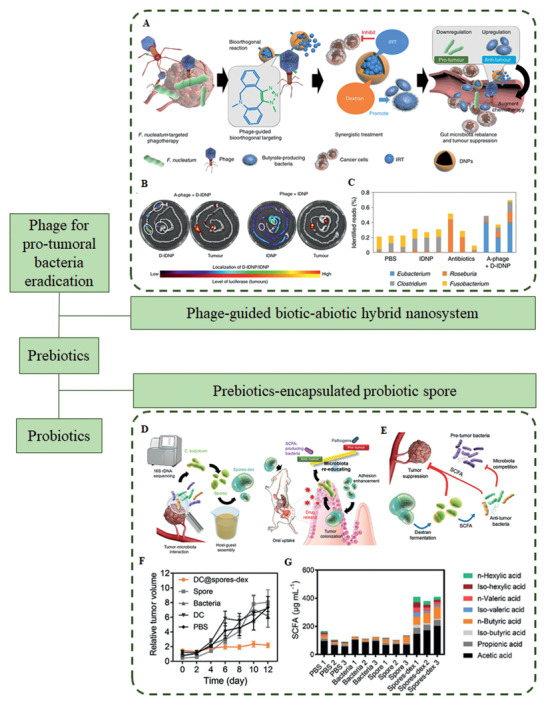
Combination cancer therapy with DDS modulating gut microbiota. A) Illustration of the phage‐guided biotic‐abiotic hybrid nanosystem and its therapeutic effects. B) Ex vivo fluorescence imaging of the intratumoral accumulation of D‐IDNPs 24 h after intravenous injection of D‐IDNPs. Images are representative of three biological replicates. Circles indicate tumors. C) Changes in gut microbiota after the treatments. The identified reads of each bacteria and total sequenced 16S rDNA were compared. Three biological replicates are shown. Sequencing was performed at day 14. Reproduced with permission.^[^ ^46^ ^]^ Copyright 2019, Springer Nature. D) Illustration of the prebiotics‐encapsulated probiotics to regulate gut microbiota and suppress colon cancer. Bacteria specifically enriching in tumor tissues were screened. *C. butyricum* was then modified with prebiotic dextran by host–guest chemistry. The system that carried the chemotherapeutic drugs was used orally by mice for colon cancer treatment. E) Schematic illustration for the possible mechanism by which spores‐dex regulate gut microbiota and suppress tumor growth. F) In vivo therapeutic effect of DC@ spore, spores, bacteria, DC, and PBS in mice bearing subcutaneous CT26 tumors. Five biological replicates are shown. G) Fecal SCFA levels after the different treatments. Three biological replicates are shown. Gas chromatography‐mass spectrometry analysis was used to analyze fecal SCFA levels at day 14 of treatments. Three biological replicates are shown. Reproduced with permission.^[^ ^211^ ^]^ Copyright 2020, Wiley‐VCH.

The first one combined chemotherapy, targeting prebiotics delivery, and pathogen elimination together. Phages have been exploited in anti‐bacterial therapy owing to their capacity to specifically lyse certain bacteria, thus can be modified into natural guides to target pro‐tumoral bacteria like *F. nucleatum*. Considering the beneficial effect of butyrate‐producing bacteria (such as *C. butyricum*),^[^ ^36^, ^46^, ^122^, ^193^ ^]^ Zheng et al. designed a hybrid nanosystem composed of azodibenzocyclooctyne‐modified irinotecan‐loaded dextran nanoparticles (D‐IDNPs) (biotic moiety), an azide‐modified *F. nucleatum*‐targeted phage (an abiotic moiety) and click chemistry‐based linkages (**Figure** **8**A).^[^ ^46^ ^]^ The nanosystem decreased the viability of *F. nucleatum* and boosted the proliferation of *C. butyricum* by dextran in vitro. In the intestinal environment, abiotic moieties acted as pre‐targets to tumor site, then the subsequently administrated D‐IDNPs linked to pre‐targets by virtue of the click chemistry reaction (Figure 8B). As a result, the hybrid nanosystem significantly inhibited the tumor growth in CT26 tumor‐bearing mice and Apc^Min/+^ mice. As observed, the decrease of *F. nucleatum* population and the flourishment of beneficial butyrate‐producing bacteria probably contributed to the anti‐tumor effects (Figure 8C). This DDS conducted comprehensive modulation on gut microbiota, and also meant that phages might be used as a kind of potential material in designing bacteria‐targeted nanosystems.

The second one combined the delivery of probiotics and prebiotics. *C. butyricum* was able to form stress‐resistant spores in CRC tumor tissues and produce the CRC‐inhibiting SCFA.^[^ ^211^ ^]^ Zhang and co‐workers chose *C. butyricum* as the probiotic moiety and prebiotic dextran, which could be metabolized into SCFA by beneficial bacteria like *C. butyricum* and *A. muciniphila*, as the carrier, constructing *C. butyricum* spores‐loaded dextran particles (spores‐dex) (Figure 8D). Dextran encapsulation enhanced the intestinal adhesion and retention capacity of spores. Spores‐dex significantly inhibited the growth and invasion of the orthotopic CT26 tumor in mice (Figure 8E), which could be attributed to the relief of microbiota dysbiosis and associated immunosuppression, as well as increased content of SCFA (Figure 8F).

### 5.4 Binding Bacteria Products with Nanoparticles

**Figure 9:**
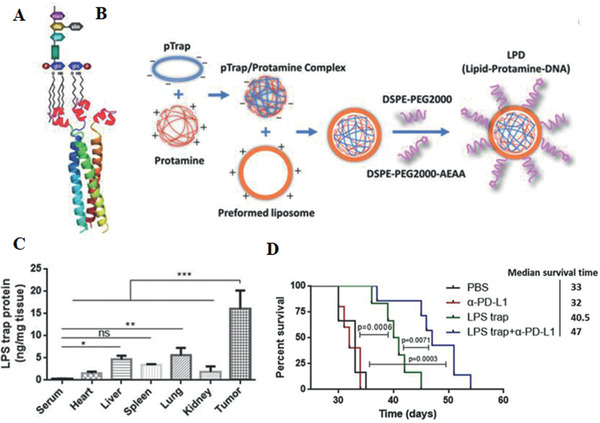
Binding bacteria products to promote immunotherapy against CRC. A) The schematic of the trimeric LPS trap and multivalent interaction of the LPS trap with the lipid A region of LPS. The red region is the LPS‐binding moiety. B) Preparation of LPS trap plasmid (pTrap)‐loaded LPD nanoparticles. C) LPS trap protein expression in serum, major organs, and CT26‐FL3 tumor. LPS trap plasmid‐loaded LPD was given on day 0, and the LPS trap expression was measured using enzyme‐linked immunosorbent assay (ELISA) on day 2 (*n* = 3). ^***^ *p* < 0.001. D) Kaplan–Meier survival curves; the difference between different groups is significant by the log‐rank test. Reproduced with permission.^[^ ^177f^ ^]^ Copyright 2018, Wiley‐VCH.

Specific products in gut microbiome play an important role in the development and progression of CRC through changing the cell cycle and normal functions. VacA secreted by *H. pylori* is a pore‐forming toxin which impairs the epithelium by inducing autophagy.^[^ ^62^ ^]^ LPS produced by Gram‐negative bacteria, as a typical activator of TLR‐4, promoted CRC development through TLR4/ MyD88/NF‐kB signal pathway.^[^ ^32^, ^56^, ^91^ ^]^ Song et al. proposed a hypothesis that LPS in the colonic environment negatively influenced CRC immunotherapy efficacy.^[^ ^177f^ ^]^ They designed a tumor‐targeting LPS‐trapping nanomedicine encapsulating the plasmid expressing a LPS‐binding fusion protein via protamine and anisamide‐modified and anisamide‐unmodified DSPE‐PEG (LPS trap) (**Figure** **9**A,B). Due to the enhanced permeability and retention effect and the positive targeting effect mediated by anisamide, the LPS‐trapping protein showed the highest expression in tumor tissues (Figure 9C). The trapping of LPS significantly increased intratumoral CD8^+^ and CD4^+^ T cells, as well as MHC II^+^ and CD86^+^ DCs. The level of CTL chemokines CXCL9 and CXCL10 was also increased in CT36‐FL3 tumors, while the PD‐L1 level was almost not changed. Therefore, administration of LPS trap efficiently improved the efficacy of anti‐PD‐L1 mAb for CRC treatment by multiple times (Figure 9D). Besides expressing bacterial products trap via transfection, nanomedicine may impede the pathogenetic adhesion of bacterial products by adsorbing them. Wang et al. developed a kind of hydrogel retaining toxin‐absorbing nanosponges. The nanosponges referred to the red blood cell membrane‐coated nanoparticles which possessed the ability to soak up the pore‐forming toxins produced by methicillin‐resistant *Staphylococcus aureus*.^[^ ^217^ ^]^ Although nanomedicine with the pro‐tumoral bacterial toxin‐blocking or toxin‐adsorbing function has not been reported, the mentioned strategies have a promising prospect in assisting cancer therapy.

### 5.5 Engineered Bacteria‐Based DDS

Bacteria have natural advantages to be functionalized as cancer therapeutic agents. First, bacteria target the tumor through complex mechanisms, including escaping through chaotic tumor vasculature, hypoxia tropism (obligate anaerobes such as *Salmonella* and *Clostridium* species as well as facultative anaerobes), self‐propulsion to penetrate the tumor tissue for acquiring energy and escaping from anti‐bacteria immune response.^[^ ^218^ ^]^ Also, some bacteria efficiently suppress tumor progression through promoting the recruitment of CD8^+^ T cells or producing anti‐tumor bacterial toxins.^[^ ^202^, ^218^ ^]^ In addition, the power of bacteria can be harnessed by genetic engineering technology or synthetic biology.^[^ ^208^ ^]^ As carriers of therapeutic agents, engineered bacteria enrich the toolkits of cancer therapy, promoting anti‐cancer immune responses solely or combining with other treatments.^[^ ^202^, ^219^ ^]^

**Figure 10:**
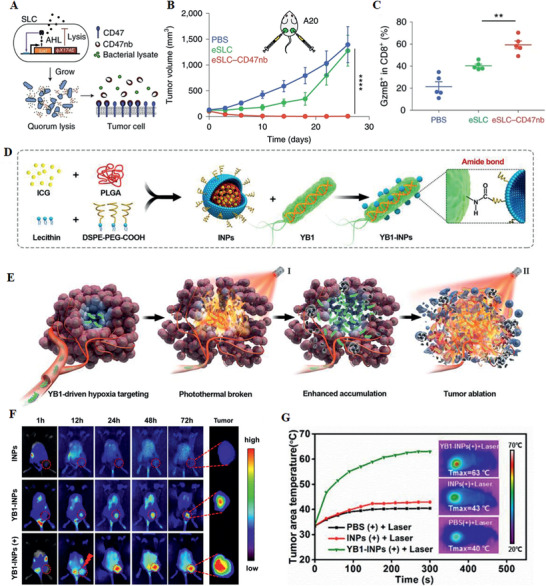
Engineered bacteria‐based DDS for cancer therapy. A) *E. coli* with SLC reach a quorum and induce the phage‐lysis protein *ϕ*X174E, leading to bacterial lysis and release of a constitutively produced, CD47‐blocking nanobody that binds to CD47 on the tumor cell surface. B) Tumor growth curves in BALB/c mice (*n* = 7 per group) bearing A20 B cell lymphoma tumors received intratumoral injections every 3–4 days with PBS, *E. coli* with SLC (eSLC) or eSLC undergoes intratumoral quorum lysis to locally release an encoded nanobody antagonist of CD47 (eSLC‐CD47nb). (^****^ *p* < 0.0001, two‐way ANOVA with Tukey's multiple comparisons test). Data are representative of two independent experimental replicates. C) Percentages of intratumoral GzmB^+^CD8^+^ T cells (*n*  =  5 per group, ^**^ *p* =  0.0011, unpaired two‐tailed *t*‐test). All data are shown as mean  ±  s.e.m. Reproduced with permission.^[^ ^212^ ^]^ Copyright 2019, Springer Nature. D) Preparation procedure of YB1‐INPs. Synthesized INPs with single‐step sonication were attached to YB1 through amide bonds. E) YB1‐INPs with hypoxia‐targeting and photothermal‐assisted bioaccumulation for tumor penetrative therapy. After migrating into tumor hypoxic cores and subsequently irradiated with NIR laser, the loosening of tumor tissue and tumor lysis generated bacteria‐attracting nutrients, which further enhanced the accumulation and coverage of YB1‐INPs in large solid tumors. Ultimately, the enriched YB1‐INPs under NIR laser irradiation completely ablated the large solid tumor without relapse. F) In vivo FL imaging of INPs, YB1‐INPs, and YB1‐INPs (+) at different time points. (+) refers to laser irradiation at 12 h for promoting bioaccumulation of YB1‐INPs. The ex vivo NIR FL images of tumors at 72 h. G) Infrared thermal images of MB49 tumor‐bearing mice exposed to laser irradiation after intravenous injection of PBS, INPs or YB1‐INPs at 72 h. Reproduced with permission. ^[^ ^213^ ^]^ Copyright 2019, Elsevier Ltd.

The ultimate goal of bacteriotherapy is transforming bacteria to smart DDS which are responsive to specific environmental stimuli, efficiently release therapeutic agents and conduct self‐elimination after the treatments finish.^[^ ^220^ ^]^ Several engineering strategies have been utilized in bacteria‐based therapies and multiple studies showed encouraging results. *Salmonella* VNP20009 without msbB and purI genes is a most studied attenuated strain of *Salmonella typhimurium* (*S. typhimurium*), which has been proven to have tumor‐targeting specificity.^[^ ^221^ ^]^ A modular synthetic adhesin containing a highly conservative *β*‐barrel domain and alterable immunoglobulin with high affinity and specificity to cancer cells has been found to endow *E. coli* with the controlled adhesion ability to tumor cells.^[^ ^222^ ^]^ In regard of cargo delivery, transferring the gene sequence of cancer therapeutic agents (e.g., cytokine, cytotoxic agents, and siRNA) or transcription regulators into bacteria is the most common modality. Probiotic EcN expressing cytotoxic agent Luminmide A from *Photorabdus luminescens* TT01 and Glidobactins from *Burkholderia* K481‐B101 reduced the tumor weights by three times compared to non‐engineered EcN.^[^ ^219a^ ^]^ Furthermore, for attenuating side effects and enhancing drug accumulation in tumors, the expression of the aimed molecules requires external signal sensing triggers at proper sites. For bacterial communities, quorum sensing takes part in transcriptional regulation.^[^ ^52^ ^]^ Autoinducers are crucial bacterial signal molecules in quorum sensing, which can be exploited as the expression switch.^[^ ^212^, ^218^, ^220^ ^]^ Chowdhury et al. engineered non‐pathogenic *E. coli* with a plasmid encoding the autoinducer luxI and a bacteriophage lysis protein (*ϕ*X174E) and another plasmid driving the constitutive expression of anti‐CD47 nanobodies (CD47nb). The engineered strain with a synchronized lysis circuit (SLC) could produce the quorum‐sensing molecule acylhomoserine lactone and express *ϕ*X174E, the increase of which resulted in the intratumoral quorum lysis of *E. coli* and the release of CD47nb (**Figure** **10**A).^[^ ^212^ ^]^ By blocking CD47, this intelligent bacteriotherapy increased tumor‐antigen‐specific CD8^+^ T cells, stimulated the rapid regression of A20 tumors, and prevented metastasis in mice (Figure 10B,C).

Conjugating drug‐loaded carriers to the surface of bacteria is another potential strategy for establishing bacteria‐based DDS. Chen et al. linked indocyanine green‐loaded PLGA nanoparticles to *S. typhimurium* YB1 (YB1‐INP), an engineered safe bacterial strain with great hypoxia‐targeting ability, through amide bonds (Figure 10D,E).^[^ ^213^ ^]^ The hypoxia‐targeting microswimmers/abiotic nanophotosensitizer hybrid system effectively accumulated in tumors and exerted photothermal effects under NIR laser irradiation (Figure 10F,G), which assisted YB1‐YNP to penetrate into tumor cores and induce a satisfactory tumor‐eradicating effect. The abundant anti‐tumor mechanisms of therapeutic bacteria enable the combination of bacteriotherapy with other cancer therapy regimens.^[^ ^208^, ^219^ ^]^ For instance, Bascuas et al. demonstrated that the immunotherapy by LVR01 (an attenuated strain of *S. typhimurium*) enhanced the efficacy of multiple chemotherapy on B‐cell non‐Hodgkin lymphoma in mice through increasing tumor‐infiltrating leukocytes and regulating the tumor microenvironment.^[^ ^219c^ ^]^ Although the safety issues such as bacterial toxicity, in time elimination of bacteria, and unstable and short‐time efficacy still exist, the engineered bacteria have potential to solely acts as therapeutic agents or be applied as great collaborators of conventional immunotherapy, chemotherapy, and radiotherapy.^[^ ^208^ ^]^

## 6 Conclusions and Outlook

On account of the impacts of gut microbiota on carcinogenesis and conventional cancer therapy including radiotherapy, chemotherapy, and immunotherapy, modern DDS regulating or based on the microbiota to ameliorate cancer therapy have become a hotly debated field. Precise microbiota modulation and satisfactory therapeutic outcomes with minimum side effects are the goals of microbial and medical science, and a number of clinical trials have been conducted in recent years. However, propelling microbiota‐based therapeutic agents and bacteriotherapy into clinical applications is still facing numerous challenges.

The human gut microbiota have coevolved with the host bodies for millions of years.^[^ ^2^, ^5^ ^]^ By virtue of high‐throughput sequence and metagenomic technologies, the diversity of gut microbiota constitution among individuals has been observed, which is influenced by multiple factors including genotype, lifestyle, ages, and diseases etc.^[^ ^1^, ^2^, ^7^, ^8^, ^9^, ^10^, ^11^, ^223^ ^]^. Since microecosystem consists of numerous microorganisms and is regulated by sophisticated signal networks, the knowledge of this delicate micro world is still limited. As a vague term, dysbiosis is not sufficient for describing the abnormal microbiota associated with various diseases, and whether it is the cause or consequence of pathological changes is unproven in most cases. In addition, depending on experimental methods and model choice, discrepant results are obtained from the studies on carcinogenesis‐associated microbiota.^[^ ^21^, ^224^ ^]^ FMT has been the most convincing and direct approach to the investigation into the relationship between abnormal bacteria and disease.^[^ ^223^ ^]^ In consideration of the limitation of human FMT, most studies used humanized gnotobiotic rodents as experimental models.^[^ ^21^, ^225^ ^]^ However, in these animals, neither can human pathogens maintain their pathogenicity, nor can the complex factors such as diet, genotype, and disease in human GIT environment be simulated.^[^ ^225^ ^]^ Therefore, although lots of studies based on humanized gnotobiotic rodents showed positive results, whether they exaggerated the causality or not is worth discussing. Walter et al. recommended several alternative experimental approaches including using more human‐like animal recipients such as non‐human primates and immunologically or metabolically ‘‘humanized’ rodents.^[^ ^224a^ ^]^ Besides, the focus of study is supposed to shift from establishing association to identifying the causality between diseases and certain bacteria and elucidating the mechanisms.^[^ ^19^, ^21^, ^224^, ^225^ ^]^

The identification of pro‐ or anti‐tumorigenesis species from complex gut bacteria communities is never an easy work, which requires elucidation about the role of the specific microbiota component in inflammation, metabolism, and tumorigenesis, or even the relationship with distal organs. Probiotics such as *Bifidobacterium* suppress exorbitant immune responses in normal mucosa to defend against chronic inflammation, which is favorable for carcinogenesis.^[^ ^73^, ^77^ ^]^ However, they also promote anti‐tumor immune responses in tumor‐bearing mice.^[^ ^40^, ^147^ ^]^ Moreover, how the quantities and viabilities of different species change during disease progression? Will the function of bacteria be influenced by physiological conditions? How the microbiota response to the intervention on single or multiple bacterial species? There are still lots of questions to be addressed.

Highly individualized microbiome indicates another challenge for the clinical application of modern DDS. Although some microbiota‐targeting DDS showed promising efficacy of tumor suppression in mice, the heterogeneity of human commensal bacteria may have unpredictable impacts on the therapeutic outcome^[^ ^10^, ^223^ ^]^. Therefore, precise diagnostics are necessary to evaluate the feasibility of different treatments through identifying cancer‐causing bacteria and detecting taxonomic profiling.^[^ ^38^, ^226^ ^]^ The combination of metagenomic analysis and diagnostic methods including electrochemical sensors, nanotechnology‐based fluorescent analysis, and bio‐barcode assays may be powerful tools for precise pathogen detection and treatment planning.^[^ ^226^ ^]^

In spite of outstanding advantages compared to traditional approaches, modern DDS for microbiota modulation are faced with considerable challenges. 1) Toxicity of carrier materials. Compared with the commonly used natural macromolecules such as polysaccharides and proteins for probiotic encapsulation, synthetic polymers and inorganic nanoparticles are less biocompatible and more health risks exist^[^ ^227^ ^]^. Inorganic nanoparticles have been found to disturb the normal gut microbiota and even lead to intestinal dysfunction.^[^ ^228^ ^]^ The organ toxicity and cytotoxicity of nanoparticles are dependent on their physico‐chemical properties and in vivo behaviors.^[^ ^227^ ^]^ More detecting technologies and models are needed to systemically study how the designing factors affect the in vivo toxicity. 2) Insufficient efficacy. In spite of less difficulties of scaling up production, simple nano‐encapsulation of probiotics only achieves limited targeting effect.^[^ ^194b^ ^]^ Although nanotechnology‐based targeting DDS have exhibited specificity for tumors, the distribution level of therapeutic agents in tumors is far from satisfactory.^[^ ^196a^ ^]^ In the case of using nanosystems for microbiota modulation, the way of various parameters of nanomedicine and individualized commensal bacterial communities influencing the therapeutic efficacy should be deeply studied. 3) Side effects and biosafety concerns. The contingent deleterious effect of modern DDS on commensal bacteria or even GIT functions must be paid more attention to. Since the therapy involves the intervention on complex microecological network, the consequence of the change in quantity and viability of both known and unknown microorganisms requires cautious preclinical and clinical evaluations as well as long‐time observation. In addition, widely used engineered bacteria may promote the transduction of DNA to intrinsic commensal bacteria, which should be considered.^[^ ^175^, ^202^ ^]^

In this review, the role of microbe and microbiota in cancer progression and cancer therapy are introduced. Then we highlight modern DDS based on microencapsulation or nanotechnology for microbiota modulating and improving cancer therapy. Several valuable works on eliminating cancer‐causing bacteria, boosting probiotic bacteria or combination of these two strategies to restore the homeostasis of bacterial communities have been demonstrated. Besides manipulating bacteria, intervention on their products is also promising. Moreover, genetic engineering and synthetic biology acting as powerful tools for developing programmable bacterial devices are discussed. Although modulating the microbiota for cancer treatment is still limited by a series of challenges, there is no doubt that with the development of material science and nanotechnology, more significant breakthroughs will be made in this burgeoning field.

## Conflict of Interest

The authors declare no conflict of interest.
